# Organocatalytic Control over a Fuel‐Driven Transient‐Esterification Network**

**DOI:** 10.1002/anie.202008921

**Published:** 2020-09-02

**Authors:** Michelle P. van der Helm, Chang‐Lin Wang, Bowen Fan, Mariano Macchione, Eduardo Mendes, Rienk Eelkema

**Affiliations:** ^1^ Department of Chemical Engineering Delft University of Technology Van der Maasweg 9 2629 HZ Delft The Netherlands

**Keywords:** acetylation, chemical reaction networks, organocatalysis, out-of-equilibrium systems, polymers

## Abstract

Signal transduction in living systems is the conversion of information into a chemical change, and is the principal process by which cells communicate. In nature, these functions are encoded in non‐equilibrium (bio)chemical reaction networks (CRNs) controlled by enzymes. However, man‐made catalytically controlled networks are rare. We incorporated catalysis into an artificial fuel‐driven out‐of‐equilibrium CRN, where the forward (ester formation) and backward (ester hydrolysis) reactions are controlled by varying the ratio of two organocatalysts: pyridine and imidazole. This catalytic regulation enables full control over ester yield and lifetime. This fuel‐driven strategy was expanded to a responsive polymer system, where transient polymer conformation and aggregation are controlled through fuel and catalyst levels. Altogether, we show that organocatalysis can be used to control a man‐made fuel‐driven system and induce a change in a macromolecular superstructure, as in natural non‐equilibrium systems.

## Graphical abstract

We report the design and study of a catalytically controlled chemical reaction network (CRN). Specifically, we designed a fuel‐driven transient‐esterification CRN, where pyridine controls ester formation and imidazole controls ester hydrolysis. This fuel‐driven strategy was further implemented in a macromolecular system, where through variation of fuel and catalysts levels, we are able to control polymer conformation and aggregation.

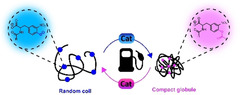

## Introduction

Signal transduction in living systems is the conversion of information into a chemical change and the primary process by which living cells are able to communicate over micrometre distances. Cells harbour very specific and sensitive signal‐transducing mechanisms, which are regulated by membrane‐bound protein receptors that respond to external signals such as antigens, light, hormones, pathogens, hypoxia, pheromones, amongst others.^[1]^ This rudimentary communication enables functions such as time keeping and signal‐amplification, which are encoded in non‐equilibrium (bio)chemical reaction networks (CRNs) regulated by enzymes.^[2]^ As such, out‐of‐equilibrium systems are an integral part of biological pathways and most complex living systems need the constant input of a chemical fuel to sustain themselves. The fuel is typically a high‐energy bond molecule, for example, adenosine triphosphate (ATP), guanosine triphosphate (GTP) or acyl‐coenzyme A (acyl‐CoA). For example, the latter fuel together with two enzymes plays a key role in histone (de)acetylation, which regulates gene activity and is recognized as a very important process in epigenetics.^[3]^ Overall, catalysis is crucial in non‐equilibrium biological CRNs and can turn ON and OFF the complete process. Inspired by catalytically controlled natural processes, we set out to design a man‐made catalytically controlled out‐of‐equilibrium network. Successful fuel‐driven CRNs have been designed before with direct (fuel integration in the building block) and indirect (fuel consumption without structure integration) chemical fuels, providing spatiotemporal control over material formation and exhibiting non‐linear behaviour, such as stochastic collapse or oscillations.^[4]^ Yet, the incorporation of catalysis as a control switch to regulate formation and degradation rates of a transient product in a non‐equilibrium CRN has only been investigated to a limited extent. Examples using enzyme catalysis in fuel‐driven CRNs exist[^2c^, ^4g^, ^4l^, ^4p^] and some recent examples of cooperative catalysis have been reported, yet those typically involve the building blocks as catalysts.^[5]^ In the current work however, we use two external catalysts in parallel to control the reaction kinetics of the CRN. Instead of enzymatic or metal‐based catalysis we turned to organocatalysis. Compared to enzymes and metal‐based catalysts, organocatalysts are frequently less active, but also simple, often less toxic, easily accessible, less substrate specific and deemed particularly useful to enhance the efficiency of chemical reaction networks.^[6]^ For applications in soft materials or otherwise crowded environments, organocatalysts also have a higher diffusive mobility and higher environmental tolerance than enzymes. Small molecule organocatalysts are used for a myriad of synthesis reactions and can be used in aqueous environment.^[6b]^ Here, we show how two organocatalysts can be used together to control the yield and lifetime of a transiently stable product in a fuel‐driven esterification CRN. First, we explain the system design and characteristics. Then, we show the performance of the system with a small molecule model CRN and investigate the reaction kinetics. Finally, we apply the same organocatalysed fuel‐driven strategy to control the shape of an amino acid functionalized polymer system by transient (de)acetylation, showing temporal regulation over polymer chain conformation and aggregation behaviour.

## Results and Discussion

### Choice of Fuel‐Driven CRN and Reaction Conditions

**Scheme 1:**
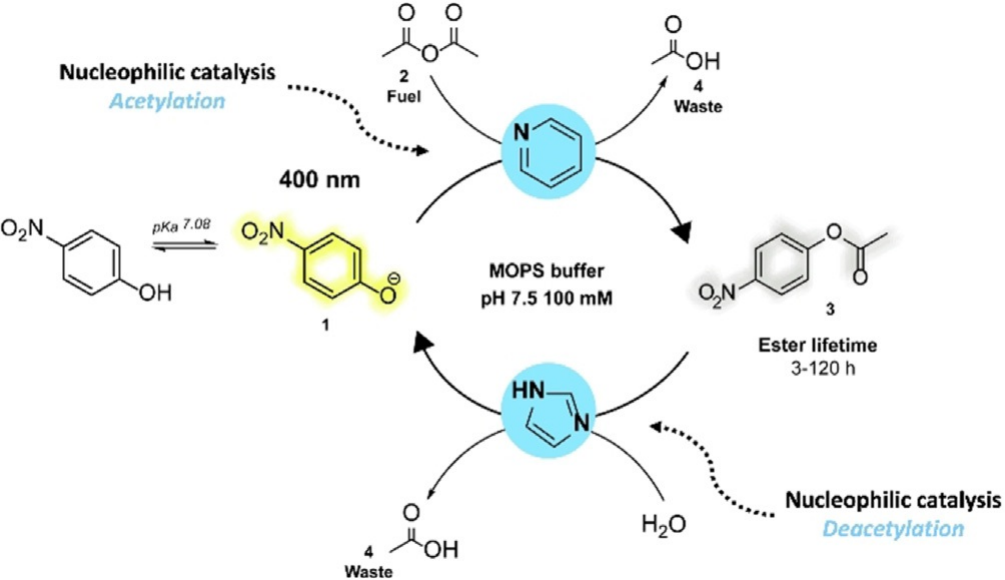
Fuel‐driven out‐of‐equilibrium esterification CRN, where the formation and degradation pathways can be controlled by different nucleophilic catalysts: the rate of ester formation is accelerated by pyridine and that of the backward ester hydrolysis by imidazole.

We designed a CRN, where the forward reaction (ester formation) can be accelerated by pyridine and the backward reaction by imidazole (ester hydrolysis) (Scheme 1). Acetic anhydride **2** is used as a direct chemical fuel, which reacts with the starting material (*p*‐nitrophenol(ate) **1**) generating the product for the transient state (*p*‐nitrophenyl ester **3**) and acetic acid as waste **4** (Scheme 1). Pyridine is a versatile tertiary amine organocatalyst, used in a plethora of everyday synthesis reactions. In particular, *O*‐acetylation is typically carried out with DMAP or pyridine as organocatalyst.^[7]^ Here, pyridine acts as nucleophilic catalyst (Lewis base—with nucleophilicity index of *N* 11.05 in water^[8]^), creating a reactive intermediate with acetic anhydride **2**: the acetyl‐pyridinium species. The p*K*_a_ of pyridine is 5.2 meaning that at neutral pH the free base species is dominant.^[9]^ In contrast, imidazole is known as an effective catalyst for the hydrolysis of activated esters such as *p*‐nitrophenyl acetate.^[10]^ The imidazole catalytic cycle proceeds through an acetyl‐imidazole reactive intermediate and the catalytic mechanism of imidazole depends on the leaving group strength of imidazole versus the ‐OR group.^[11]^ Esters with weak leaving groups are subject to general base catalysis, whereas for activated esters with better leaving groups (here: nitro‐phenolates) the imidazole catalysis exhibits a nucleophilic substitution mechanism.[^10a^, ^10c^] Additionally, the nucleophilic catalysis of imidazole (p*K*_a_ 6.9) is pH‐dependent and favours higher pH, since more free base is present.^[12]^ Imidazole has a higher basicity than pyridine, yet the nucleophilicity is lower (*N* 9.63 in water^[13]^). Overall, imidazole has a preference for less reactive acylation agents (activated esters) compared to pyridine, which is a better catalyst for highly reactive acylation agents (anhydrides).^[14]^ The latter is exploited in this fuel‐driven CRN to control the temporal acetylation of *p*‐nitrophenol(ate) **1**. Experiments are performed at near neutral pH 7.5 in a strongly buffered system to take care of the acid waste. At pH 7.5 the phenolate is dominant over the phenol form, and the uncatalysed ester and anhydride hydrolysis are less prevalent than at more acidic or basic conditions.

### Organocatalysed Fuel‐Driven Esterification CRN

**Figure 1:**
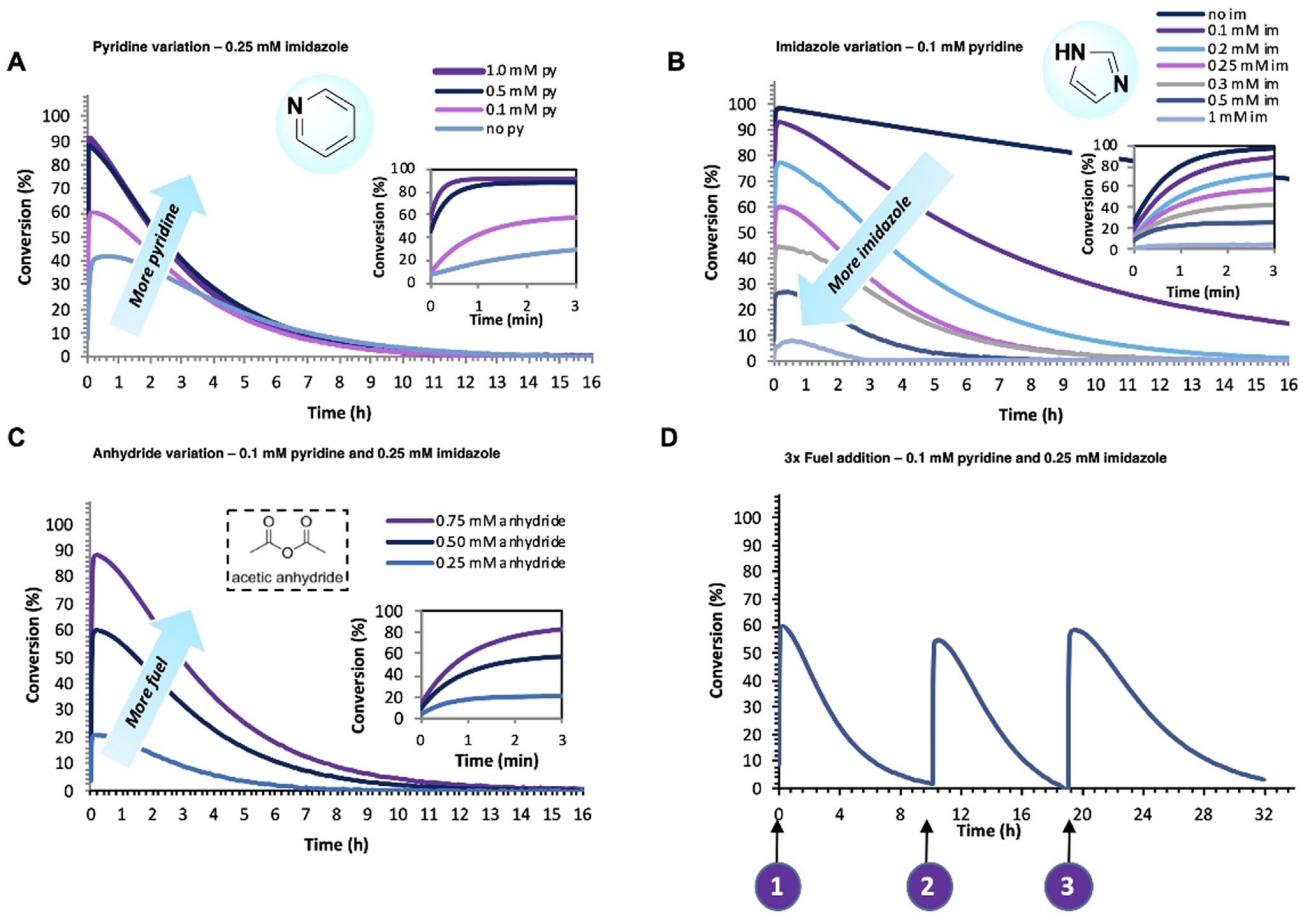
Fuel‐driven esterification network controlled by organocatalysts pyridine and imidazole in MOPS buffer (100 mm, pH 7.5) with 5 % acetonitrile. The conversion of *p*‐nitrophenol(ate) (**1**) is monitored by UV/Vis spectroscopy, following the absorbance at 400 nm over time. A zoomed‐in region of the first 3 min (0.05 h) of the reaction is shown for the pyridine, imidazole, and anhydride variation plots. A) Pyridine variation: 0.1 mm *p*‐nitrophenol (**1**), 0.5 mm acetic anhydride (**2**), 0.25 mm imidazole, and varying 0–1.0 mm pyridine. B) Imidazole variation: 0.1 mm *p*‐nitrophenol (**1**), 0.5 mm acetic anhydride (**2**), 0.1 mm pyridine and varying 0–1.0 mm imidazole. C) Acetic anhydride (**2**) variation: 0.1 mm *p*‐nitrophenol (**1**), varying 0.25–0.75 mm acetic anhydride (**2**), 0.25 mm imidazole and 0.1 mm pyridine. D) Three consecutive fuel cycles: 0.1 mm *p*‐nitrophenol (**1**), 0.5 mm acetic anhydride (**2**), 0.25 mm imidazole, and 0.1 mm pyridine. The second and third cycles were initiated by addition of a new batch of fuel **2**.

In this CRN, *p*‐nitrophenol(ate) **1** is acetylated with acetic anhydride **2** to generate ester **3**, which is hydrolytically unstable and over time the phenol(ate) **1** is regenerated along with forming acetic acid waste product **4**. Pyridine and imidazole are used as organocatalysts to accelerate the ester formation and hydrolysis. We varied the organocatalysts and fuel concentrations to achieve yield and lifetime variations in the transient *p*‐nitrophenyl ester **3** (Figure 1—shows the conversion of **1** monitored by UV‐VIS at 400 nm). While a blank reaction without organocatalysts resulted in ester lifetimes of ≈68 h and a max. yield of 55 % after 25 min. (SI Figure S7), increasing the pyridine concentration accelerated the forward reaction (Figure 1 A—zoomed‐in region of first 3 min) and augmented the overall ester yield (Figure 1 A). Supply of more pyridine than anhydride **2** (0.5 mm) however did not give any effect in yield and only a very minor increase in the forward reaction by reaching the plateau‐level a few seconds earlier as shown in Figure 1 A. This observation supports the nucleophilic catalytic mechanism for pyridine, reacting directly with the anhydride and forming the reactive acetyl‐pyridinium intermediate. Similarly, the ester degradation could be very precisely controlled with imidazole (Figure 1 B). When imidazole is absent, the ester is stable for over 120 h (with a yield close to 100 % after 25 min), whereas increasing the imidazole concentration leads to lower ester yields and shorter lifetimes, varying from 3 to 120 h. The amount of fuel provides another way to control the ester formation and lifetime. Logically, addition of more fuel results in higher ester yields and lifetimes as shown in Figure 1 C. On top of that, multiple fuel cycles could be performed sequentially (Figure 1 D). Fresh fuel was added for three consecutive times and the reaction cycle was completely repeatable and robust.

HPLC‐MS data confirmed the formation and degradation of **3** in the fuel cycle (SI Figures S8–12). Additionally, monitoring the colour change of the fuel cycle over time (SI Figure S13) proves the successful formation (yellow to colourless solution) and subsequent degradation of the ester (colourless to yellow solution), while the pH is stable (SI Figure S14). Nonetheless, the drawbacks of this system are the rapid hydrolysis of the acetic anhydride **2** fuel in water (hydrolysis rate constant 0.1575 min^−1^ ^[15]^), resulting that after 30 min only the backward reaction remains. Also, the generation of twice as much acetic acid as waste limits the amount or number of fuel additions and calls for a strongly buffered system. Therefore, other activated carboxylic acids (i.e. vinyl acetate, isopropenyl acetate) were also tested. Yet, they were not efficient (>500 fuel equivalents were required) and besides did not dissolve well in the water phase (SI Figure S15 and Table S1).

**Figure 2:**
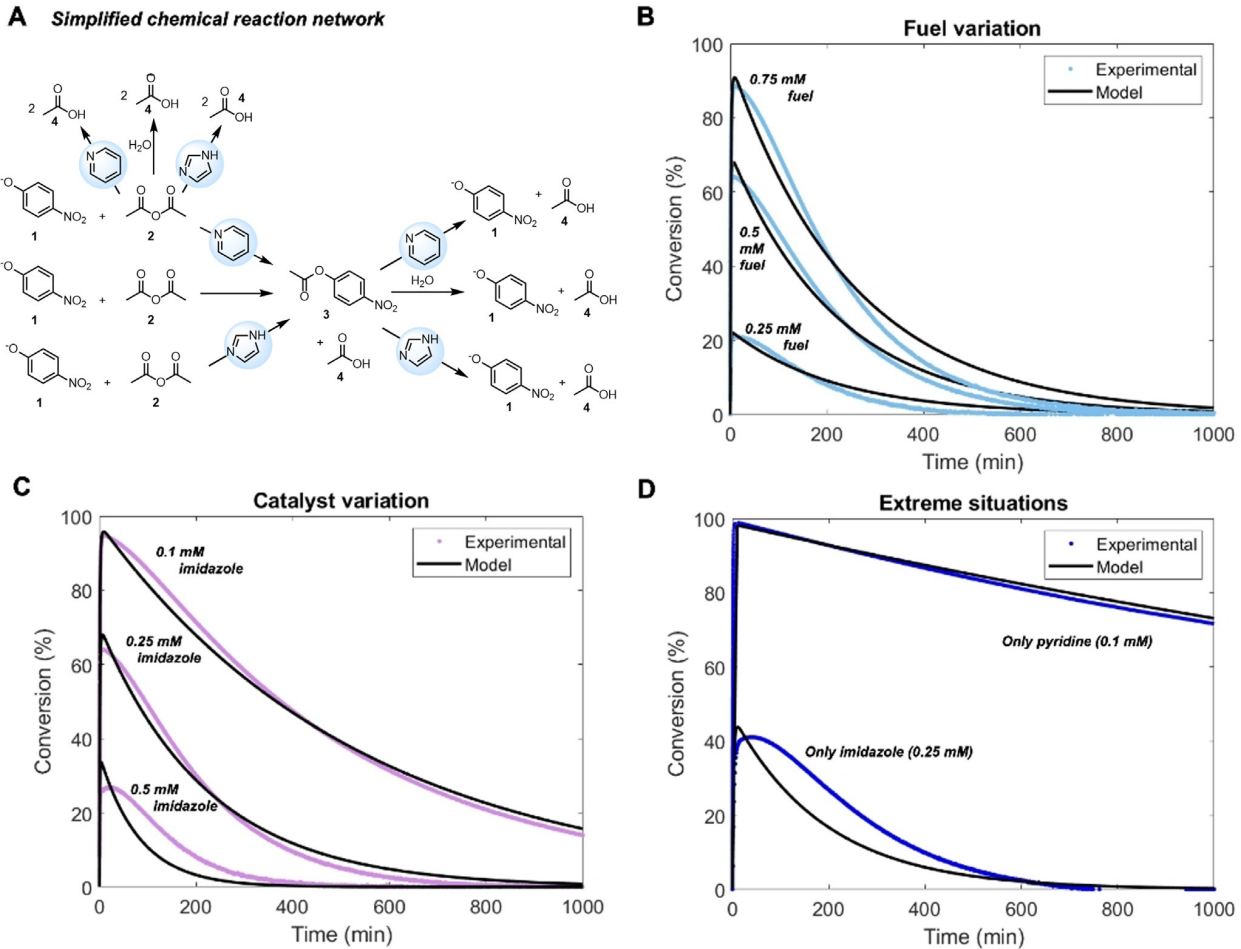
Experimental and model data comparison for varying organocatalysts and fuel concentrations, showing the conversion of *p*‐nitrophenol(ate) (**1**; start: 0.1 mm) in MOPS buffer (100 mm, pH 7.5) with 5 % acetonitrile. A) Simplified chemical reaction network with pyridine and imidazole catalyzing ester formation, ester hydrolysis, anhydride hydrolysis, and blank reactions. B) Fuel variation: 0.25/0.5/0.75 mm acetic anhydride (**2**), 0.25 mm imidazole, and 0.1 mm pyridine. C) Catalyst variation: 0.5 mm acetic anhydride (**2**), 0.1/0.25/0.5 mm imidazole, and 0.1 mm pyridine. D) Extreme situations: 0.5 mm acetic anhydride (**2**), 0/0.25 mm imidazole and 0/0.1 mm pyridine. The presented experimental data are equal to Figure 1. Details of the kinetic model are provided in the Supporting Information.

In order to understand the underlying reaction mechanisms and the role of the catalysts in the rate accelerations, a kinetic model was developed and numerically solved with Matlab software (see SI for full explanation, reaction equations and mechanism). In this simplified model it was assumed that both pyridine and imidazole catalyse the ester formation, hydrolysis and anhydride hydrolysis (Figure 2 A). The preference for imidazole for ester hydrolysis and pyridine for ester formation is expressed in the value of the rate constants. The imidazole catalysed ester hydrolysis rate constant was determined experimentally and all other constants were taken from the literature (both used as initial guesses) or optimized by the kinetic model with experimental data input. In Figure 2 (and SI Figures S30–32) the experimental data are compared with the modelled data. The model predicts the experimental data accurately for varying fuel (Figure 2 B) and organocatalyst concentrations (Figure 2 C). Also, in extreme situations where only one of the catalysts is present, the model prediction with the simplified CRN matches with the experiments (Figure 2 D). Only for high imidazole concentrations the model starts to deviate from the experimental data (Figure 2 C and SI Figure S31F). We expect that this deviation is due to the presence of the acetyl‐imidazole species, which is not consumed immediately, and hence the experimental data show a more gradual decay than the model. In this simplified model, it was assumed that the reactive intermediates are unstable and hence were not taken into account.

Furthermore, when comparing the reaction rates for ester **3** formation and degradation (Figures S33,34) it becomes clear that the formation reaction vanishes quickly (Figure S34—after 10–20 min), around the time when most of the fuel **2** is consumed, while the backward reaction is active for hours. Imidazole is responsible for an increase in the backward reaction rate (Figures SI 33A,B and 34A,B), while in the presence of pyridine the forward reaction rate is increased (Figure S34A,C—steeper slopes) and anhydride **2** is consumed faster. Within the limits of the model, transient nitrophenol acetylation is accurately described by the model, corroborating the proposed simplified catalytic CRN. Also, it allows prediction of ester yield and lifetime based on the catalyst input.

### Fuel‐Driven Responsive Polymer System

**Figure 3:**
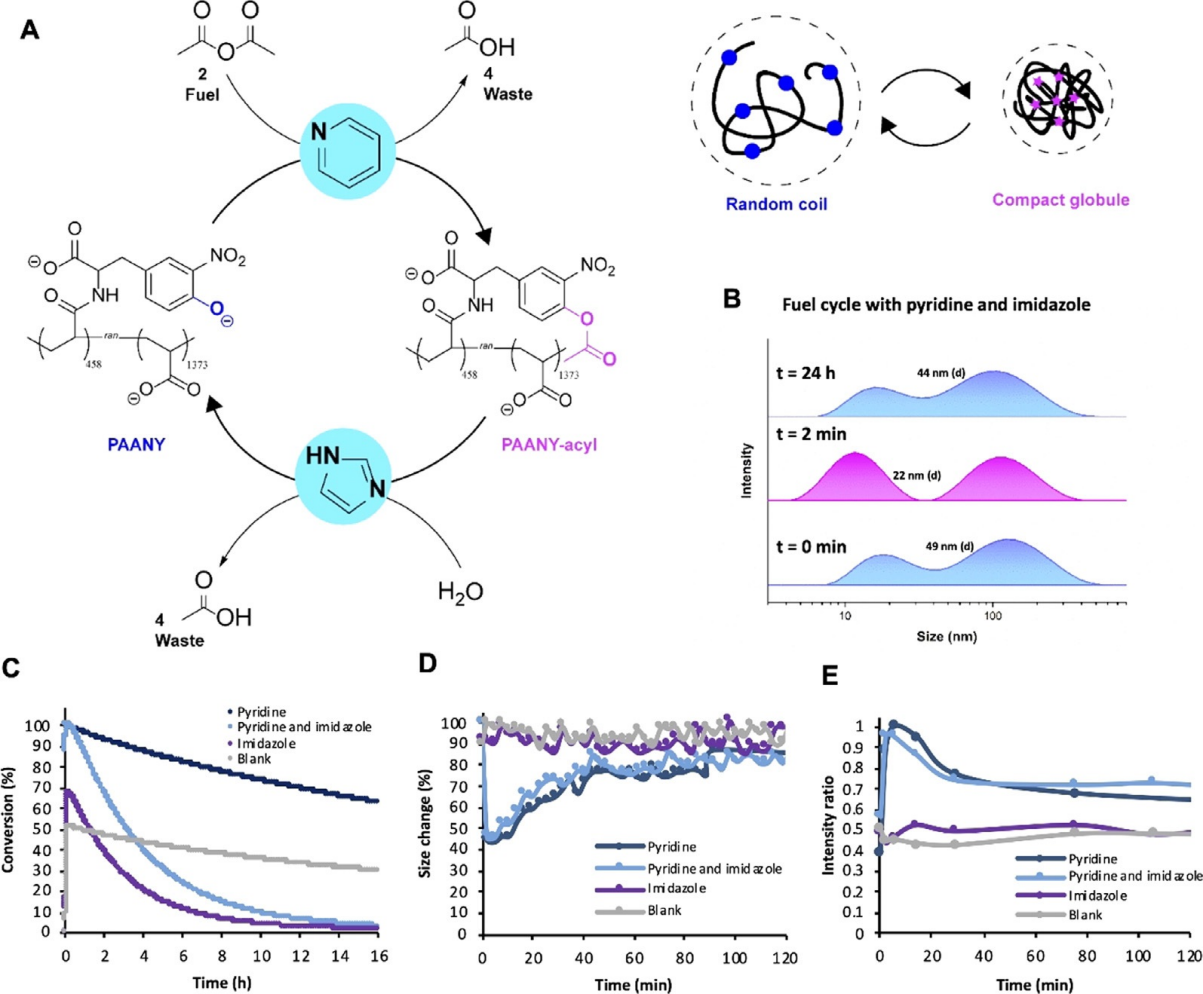
Fuel‐driven responsive polymer system controlled by organocatalysts pyridine and imidazole in borate buffer (200 mm, pH 8.0). A) Schematic overview of transient acetylation of poly(acrylic acid) 3‐nitro‐L‐tyrosine (PAANY) to PAANY‐acyl by acetic anhydride (left) and the random coil to compact globule transition (top right). B) DLS size distributions by intensity over time for 0.30 mm PAANY (0.24 mg mL^−1^), 6 mm acetic anhydride (**2**), 0.75 mm imidazole, and 0.30 mm pyridine. C) The conversion of PAANY is monitored by UV/Vis spectroscopy, following the absorbance at 420 nm over time. Conditions: 0.15 mm PAANY (0.12 mg mL^−1^), 3 mm acetic anhydride (**2**), 0–0.375 mm imidazole and 0–0.15 mm pyridine. D) Polymer size change monitored over time based on *z*‐average size (nm) measured with DLS. E) Intensity ratio of single polymer chain versus aggregate as a function of time calculated from the peak areas of the unimer and the aggregate. Conditions: 0.30 mm PAANY (0.24 mg mL^−1^), 6 mm acetic anhydride (**2**), 0–0.75 mm imidazole, and 0–0.30 mm pyridine. See Figures S38, S39 in the Supporting Information for corresponding DLS size distributions.

Having established a CRN in which the formation and degradation pathways can be controlled by two organocatalysts, we then applied this CRN to a macromolecular system. The importance of catalytic control in fuel‐driven macromolecular systems is also witnessed in chromatin condensation, a process that regulates the accessibility of DNA for transcription. The DNA (un)binding to histone proteins due to fuel‐driven transient (de)acetylation of lysines in the amino‐terminal tails of histone proteins is regulated by two enzymes and changes the nucleosome superstructure.^[3]^ In analogy, with catalytically controlled fuel‐driven acetylation of a polymer, we aim to control transient charge density and in that way polymer chain conformations, such as shape and hydrodynamic radius (Figure 3 A). In our system, a nitrophenol amino acid analogue, 3‐nitro‐l‐tyrosine, was grafted onto a 130 kDa poly(acrylic acid) (PAA) backbone via peptide coupling, giving PAANY (poly(acrylic acid) 3‐nitro‐l‐tyrosine) with 25 % NY coverage (see SI for synthetic details and characterization). PAA of relatively high molecular weight is known to have a shape transition from random coil to compact globule upon pH change due to (de)protonation of the acetate groups.^[16]^ Based on previous reports on transient polymer assembly,^[17]^ we hypothesized that such coil to globule shape transitions can be induced by transient acylation leading to a decrease of charge and reduced repulsion, and an increase in hydrophobic surface. Successful fuel cycles of the PAANY polymer with pyridine and imidazole catalysis were confirmed by UV‐VIS experiments by following the absorbance of 3‐nitro‐l‐tyrosine at 420 nm in borate buffer (200 mm, pH 8.0) (Figure 3 C). Borate buffer at pH 8.0 was chosen because of its good solvation properties for the functionalized polymer. At pH 8.0 a larger proportion of negatively charged phenolates (89 %) is present compared to pH 7.5 (72 %), potentially facilitating a larger effect on polymer conformation upon acetylation. Next, the polymer size transition in a fuel cycle was investigated with dynamic light scattering (DLS) (Figure 3 B,D). The size change in *z*‐average diameter was monitored, which includes both single chain polymers (≈10 nm) and polymer aggregates (≈100 nm) (Figure 3 B and SI Figure S38,39). The aggregate formation is most likely caused by borate interacting with carboxylate groups on the PAA backbone and carboxylate and phenolate anions on the NY functionalities. Boric acid or borate anions are known to form polymer hybrid structures due to hydrogen bonding. At neutral pH boric acid can even react with carboxylate groups to form a boric anhydride crosslink.^[18]^ The aggregate formation in borate is not specific to functionalized PAA, because it is also observed for unmodified PAA in borate buffer (SI Figure S40).

**Figure 4:**
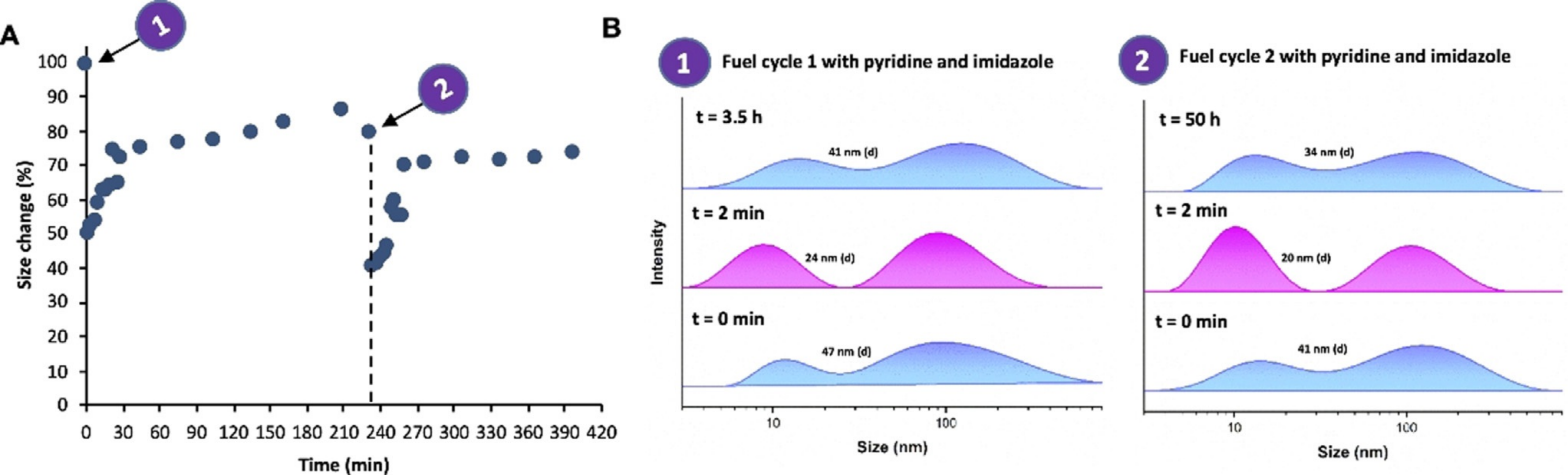
Two consecutive fuel cycles for the fuel‐driven responsive polymer system controlled by organocatalysts pyridine and imidazole in borate buffer (300 mm, pH 8.0). A) Polymer size change monitored over time based on *z*‐average size (nm) measured with DLS. B) DLS size distributions by intensity over time. Conditions: 0.30 mm PAANY (0.24 mg mL^−1^), 6/12 mm acetic anhydride (**2**), 0 0.75 mm imidazole, and 0–0.30 mm pyridine. The second cycle was initiated by addition of fresh fuel **2**.

With pyridine present, upon addition of fuel **2** the hydrodynamic radius quickly reduces by more than 50 % after which it slowly comes back to almost the original size (Figure 3 D and S38,39). We expect that the radius does not fully come back to the original value due to a slight drop in pH (from 8.0 to 7.7). In detail, the *z*‐average diameter starts at 49 nm, goes to 22 nm (at *t*=2 min) and back to 44 nm after 2 h, while the number average goes from 8.6±2.6 nm to 5.9±1.6 nm (at *t*=2 min) back to 7.4±2.0 nm. Concomitantly, the ratio between the two peaks (peak area) in the DLS size intensity distribution changes (Figure 3 B and SI Figures S38A,B). At the start the aggregate peak around 100 nm is more prevalent than the single polymer chain (unimer) peak around 10 nm, whereas after fuel addition (at *t*=2 min) the unimer peak has a larger contribution, which changes again at the end of the fuel cycle. Based on this finding, it becomes clear that the fuel‐driven acetylation changes the portion of polymers present as single chains (unimers) and in an aggregated form. The unimer to aggregate ratio as a function of time based on the peak areas is depicted in Figure 3 E. Similar to the change in the *z*‐average diameter over time, we can identify a transient shift towards the unimer upon fuel addition and acetylation in the presence of pyridine. Hence, the transient acetylation shifts the unimer/aggregate equilibrium and after 2 h the equilibrium is restored again. This equilibrium shift could indicate that the non‐equilibrium compact polymer structure (when acetylated) has a preference for the unimer over the aggregate, whereas the equilibrium random coil structure is rather in the aggregated form. However, when only imidazole or no catalysts (blank reaction) are present, no significant size change and no aggregate to unimer shift is observed (Figure 3 D,E and SI Figures S38–39C,D). By comparing the DLS size data with the UV/Vis conversion (Figure 3 C) for the measurements with imidazole and the blank it becomes clear that the compact polymer structure expands to a coil after 2 h, with about 70 % of conversion as a threshold value, meaning about 60 % of phenolate (negative charge) needs to be acetylated to induce a polymer conformational change. Based on this threshold criterion and by looking at the UV‐VIS conversion result (Figure 3 C), the polymer sample with only pyridine should collapse after 16 h, but in Figure 3 D, we observe a faster decay. We hypothesize the behaviour of the polymer chain in solution is more complex and the conversion kinetics and the polymer shape transition have a non‐linear relationship. Similar to the small molecule CRN, consecutive fuel cycles could be performed with PAANY by addition of a new batch of fuel (Figure 4).

**Figure 5:**
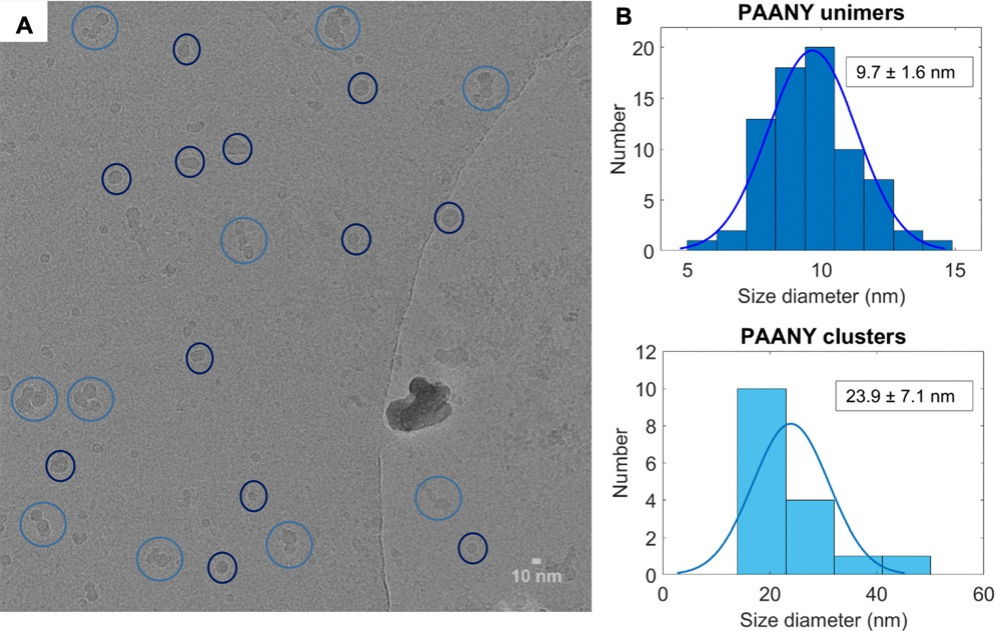
Cryo‐EM measurement of PAANY in borate buffer (200 mm, pH 8.0). A) Representative cryo‐EM image of PAANY, where the dark blue circles represent spherical polymer structures and the light blue circles the polymer clusters. B) Histograms of PAANY unimers (top) with average diameter of 9.7±1.6 nm and PAANY aggregates (bottom) with average diameter of 23.9±7.1 nm, both calculated from multiple cryo‐EM images.

The *z*‐average diameter change (Figure 4 A) and the size distributions (Figure 4 B) show similar trends as with the single fuel cycle, having a sharp size change directly after fuel addition and shift in the intensity distribution to ≈10 nm. Because of the addition of more anhydride and hence more acid waste production, the pH drops from 8.0 to 7.5, even though a buffer of 300 mm was used to mitigate this pH drop. Hence, the final size of the polymer is 34 nm and does not come back to the original value (47 nm). Moreover, the true hydrodynamic radius of the single polymer chain was confirmed by DOSY NMR diffusion coefficient measurements^[19]^ using the Stokes Einstein relation, giving similar sizes in the nanometre range (≈8 nm) as with DLS (SI Figure S42) and in good agreement with earlier literature examples on PAA polymers.^[20]^ The polymer in borate buffer was also imaged with cryo‐EM, revealing globular structures with an average diameter of 9.7±1.6 nm along with larger clusters with an average largest dimension of 23.9±7.1 nm (Figure 5 and S43). The 10 nm structures are in line with the DLS and DOSY single polymer diameter, yet the diameter of the polymer aggregates in cryo‐EM appears somewhat lower than the 100 nm observed with DLS. There, it might be that the polymer aggregate diameter from DLS is overestimated, as DLS measures fractal dimensions.

Because of the conformational change from coil to compact globule for PAANY upon acetylation by chemical fuel **2** we would expect to observe a viscosity change during the cycle. Despite the noisy data typical of dilute solutions in a rheology measurement, time sweep measurements (SI Figure S44) showed a lowered viscosity immediately after acetylation of PAANY. On a timescale of hours, the viscosity of acetylated PAANY changes to larger values (Figure S44B), which is in line with the DLS results. Control experiments on buffer, PAANY or where fuel was added to buffer, do not show any significant viscosity change (SI Figure S44A,C,D). Furthermore, with additional flow step measurements we did not observe a change in the behavior of the polymer solution (acetylated or not) compared to the buffer and observe a shear‐thinning behavior for all samples (SI Figure S45). In contrast to the time sweep measurements, the flow step measurements probe the behavior of the bulk solvent, which is similar for all.

Altogether, transient polymer conformational and aggregation changes of PAANY were achieved with this fuel‐driven esterification CRN for which the presence of the pyridine catalyst turned out to be critical. Yet, the exact behaviour of the functionalized polyelectrolyte in response to organocatalytic action is more complex than anticipated.

## Conclusion

In this work, we have shown how two organocatalysts can be incorporated in a fuel‐driven esterification CRN and individually regulate product yield and lifetime. In this CRN, *p*‐nitrophenol(ate) is acetylated with acetic anhydride as a chemical fuel, and pyridine and imidazole are used as organocatalysts to accelerate the forward and backward reaction, respectively. Variation of the chemical fuel and organocatalysts concentrations enabled full control over ester yield and lifetime. A kinetic model was developed, which corroborated the experimental results. As a proof of principle, the same organocatalytic control was applied on an amino acid functionalized polymer system, leading to control over polymer chain conformation in time. Overall, we have demonstrated that organocatalysis is a powerful tool to regulate reaction kinetics of a non‐equilibrium CRN and with that temporally control material properties by inducing a change in a macromolecular superstructure, reminiscent of living systems.

## Conflict of interest

The authors declare no conflict of interest.

## Supporting information

As a service to our authors and readers, this journal provides supporting information supplied by the authors. Such materials are peer reviewed and may be re‐organized for online delivery, but are not copy‐edited or typeset. Technical support issues arising from supporting information (other than missing files) should be addressed to the authors.

Supplementary
