# Supplementary material for: Organocatalytic Control over a Fuel‐Driven Transient‐Esterification Network
Source: Angew Chem Int Ed Engl. 2020 Sep 2;59(46):20604–11. doi: 10.1002/anie.202008921 (PMC7693295; doi:10.1002/anie.202008921)
Supplement: Supplementary file 1 — Supplementary [file ANIE-59-20604-s001.pdf]

Angewandte Chemie
Eine Zeitschrift der Gesellschaft Deutscher Chemiker

Supporting Information

Organocatalytic Control over a Fuel-Driven Transient-Esterification Network**

*Michelle P. van der Helm, Chang-Lin Wang, Bowen Fan, Mariano Macchione, Eduardo Mendes, and Rienk Eelkema**

anie_202008921_sm_miscellaneous_information.pdf

Table of Contents

1 Experimental details 2
1.1 General materials and methods 2
1.2 Polymer functionalization: 3-nitro-L-tyrosine on poly(acrylic acid) PAANY 2
1.3 UV-Vis assay 3
1.4 DLS measurements 3
1.5 Viscosity rheology measurements 4
1.6 Kinetic modelling 4
2 UV-VIS spectroscopy 4
2.1 Calibration lines 4
2.2 Absorbance plots 5
3 Fuel cycle error analysis 6
4 Blank reaction without catalysts 7
5 HPLC-MS analysis 8
6 Monitoring esterification network by color progress 13
7 pH monitoring of fuel-driven esterification network 15
8 Different acyl donors 16
9 Kinetic model for fuel-driven esterification CRN 17
9.1 Blank reaction 18
9.2 Pyridine catalysis and blank reaction 20
9.3 Imidazole catalysis and blank reaction 22
9.4 Pyridine and imidazole catalysed reaction cycle 25
9.4.1 Pyridine variation: experimental data versus model 32
9.4.2 Imidazole variation: experimental data versus model 33
9.4.3 Acetic anhydride variation: experimental data versus model 34
9.4.4 Explanation for model optimization and deviations 35
9.4.5 Ester forward and backward reaction rate comparison 36
10 FTIR PAANY 38
11 ^{1}H NMR PAANY 39
12 DLS size distributions 40
13 DLS size calibration 42
14 DOSY diffusion coefficient and size calculation 43
15 Cryo-EM imaging of PAANY 44
16 Viscosity measurements of PAANY 45
References 47

1 Experimental details

1.1 General materials and methods

Chemicals were purchased in the highest available purity and used without further purification unless reported otherwise. *p*-Nitrophenol **1**, acetic anhydride **2**, pyridine anhydrous, imidazole, isopropenyl acetate, vinyl acetate, poly(acrylic acid) (PAA ~130 kDa), N,N-dimethyl formamide (DMF) anhydrous, dimethyl sulfoxide (DMSO), N-(3-Dimethylaminopropyl)-N′-ethylcarbodiimide hydrochloride (EDC), acetonitrile, N,N-diisopropylethylamine (DIPEA), were purchased from Sigma-Aldrich. *p*-Nitrophenyl acetate **3a** was from TCI Europe. 3-nitro-L-tyrosine ethyl ester (NY-ethyl ester) was from Chem Impex International. 1-Hydroxybenzotriazole hydrate (HOBt) was from Acros Organics. Solid salts were used for the preparation of aqueous buffers: sodium tetraborate decahydrate (Borax), boric acid, sodium hydroxide were from Sigma Aldrich and 3-(N-Morpholino)propanesulfonic acid (MOPS) from Alfa Aesar. Unless stated otherwise, all preparations and analyses were performed at room temperature (RT) (~21 °C) and atmospheric pressure. Nuclear Magnetic Resonance (NMR) experiments were performed using Agilent-400 MR DD2 (400 MHz for ^{1}H and 100.5 MHz for ^{13}C) at 25 °C using residual deuterated solvent signals as internal standard. To suppress the water peak, PRESAT configuration (suppress one highest peak) was used. Prior to every diffusion-ordered spectroscopy (DOSY) NMR measurement, scouting experiments were performed to obtain the longest t1 relaxation and get an optimal diffusion gradient and delay. DOSY spectra were analysed with MestReNova software. UV-Vis spectroscopic experiments were carried out using Analytik Jena Specord 250 spectrophotometer; quartz cuvette with a 1 cm path length, volume of 3 mL, at RT. Liquid Chromatography–Mass Spectrometry (LC-MS) was performed on a Shimadzu Liquid Chromatograph Mass Spectrometer 2010, LC-8A pump with a diode array detector SPD-M20. Negative and/ or positive mode Electro Spray Ionization Mass Spectrometry (ESI-MS) was used for the peak assignment. Fourier Transform Infrared Spectroscopy (FTIR) spectroscopy was performed with NicoletTM 6700 FT-IR Spectrometer from Thermo Electron Corporation equipped with OMNIC Software using the ATR method. Spectra were recorded at wavenumber range 4000-400 cm^{-1} with 4 cm^{-1} resolution. Prior to each experiment a background of the ZnSe crystal was measured. Dynamic light scattering (DLS) measurements were recorded with a Malvern Zetasizer Nano equipped with a 633 nm laser, collecting the optical data with a 90° scattering angle; quartz cuvette with 1 cm path length, volume of 3 mL, at 20 °C. Cryo-EM (electron cryo-microscopy) images were obtained with a JEOL JEM3200-FSC, operated at 300kV with Gatan camera K2-Summit operated in counting mode with 10s exposure time, dose fractionated 0.2s aligned by SerialEM. Zero-Loss filtered with a 20eV slit. Samples were prepared with a Leica plunger EM GP, 21 °C, 98% RH, 8 seconds blotting time. The final images were analysed with ImageJ software. Polymer viscosity change was measured using a rheometer (AR G2, TA instruments) equipped with a steel plate-and-plate geometry of 40 mm in diameter, using a with hexadecane filled water trap.

1.2 Polymer functionalization: 3-nitro-L-tyrosine on poly(acrylic acid) PAANY

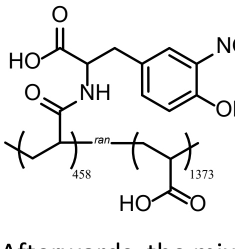

PAANY

Poly(acrylic acid) PAA (M_n 130-230 kDa (reported by Sigma-Aldrich), Đ = 2.3 (GPC in water)) was functionalized with 3-nitro-L-tyrosine (NY)-ethyl ester via peptide coupling with EDC/HOBt and DIPEA in DMF. For 25% coverage: PAA (50 mg) was mixed with NY-ethyl ester (50 mg, 0.25 molar eq.), EDC (50 mg, 0.5 molar eq.), HOBt (102.3 mg, 0.625 molar eq.) and DIPEA (138 µL, 1.125 molar eq.) in DMF (15 mL). The reaction mixture was stirred for 24 h at RT. Afterwards, the mixture was concentrated under reduced pressure and the final product precipitated in a

DMSO/ water mixture, giving PAANY-ethyl ester (yield: 79%). To obtain the hydrolysed polymer PAANY, stock solutions were left overnight in the respective buffer solution and the hydrolysis was confirmed by ^{1}H NMR (Figure S37). **^{1}H NMR** (400 MHz, DMF): δ = 12.61 (s, 1H, -OH), 10.93 (s, 1H, -COOH), 7.88 (s, 1H, -ArH-), 7.57 (s, 1H, -ArH-), 7.17 (s, 1H, -ArH), 4.64 (s, 1H, -CHα-), 4.11 (s, 2H, $-CH_2-$), 3.12 (s, 2H, -CHβ-), 2.00-1.50 (PAA backbone), 1.17 (s, 3H, $-CH_3$) . N.B. the multiplicity of 3-nitro-L-tyrosine in the NMR spectrum is lost because the peaks are too broad on the polymer (appearance as broad singlets). After hydrolysis the peaks of the ethyl ester at 4.11 and 1.17 ppm have disappeared. **FTIR** (ATR, cm^{-1}): ν 1730 (C=O stretch ester), 1700 (C=O stretch acid backbone), 1680 (C=O stretch amide), 1350 (OH-bend phenol), 1050 (C-O-C stretch ester).

1.3 UV-Vis assay

Stock solutions were prepared in MOPS buffer (pH 7.5, 100 mM), borate buffer (pH 8.0, 200 mM) or acetonitrile (for acetic anhydride **2** only – to avoid significant hydrolysis in the stock solution). Unless stated otherwise, the fuel cycle was performed with 0.1 mM *p*-nitrophenol **1**, 0.5 mM acetic anhydride **2**, 0-1 mM of pyridine, and 0-1 mM of imidazole in MOPs buffer (pH 7.5, 100 mM), in quartz cuvettes, path length of 1 cm (total reaction volume of 3 mL) at RT. The stock solutions of the reactants were always added in the following order: *p*-nitrophenol **1**, pyridine, imidazole and acetic anhydride **2**. Teflon caps were used to close the cuvette. The cuvette was turned upside down to mix the solution. The reactant peak was followed using slow time scan, measuring wavelength 400 nm. The pH was measured before and after the reaction (or followed during the reaction; see SI: pH monitoring of fuel-driven esterification network). The conversion was calculated with the extinction coefficients (see SI for calibration lines) and Lambert-Beer law:

$$A = \varepsilon l C$$

, where A is the absorbance, ε the extinction coefficient, l the path length of the cuvette and C the concentration. The experiments with PAANY were performed similarly, only following the decrease/ increase at 420 nm from NY. Because we anticipated problems with concentrated sulfonate buffers with macromolecules (such as precipitation), we used borate buffer (pH 8.0, 200 mM). Experiments were performed at pH 8.0 to have a higher percentage of phenolate (negative charge) compared to phenol and co-solvent was avoided, since it could affect the polyelectrolyte behaviour in solution.

1.4 DLS measurements

Stock solutions were prepared in borate buffer (pH 8.0, 200 mM) and filtered with syringe filters (0.2 µm) before use. Unless stated otherwise, the fuel cycle was performed with 0.30 mM PAANY (0.24 mg/mL), 6 mM acetic anhydride **2**, 0-30 mM of pyridine, and 0-0.75 mM of imidazole in borate buffer (pH 8.0, 200 mM), in quartz cuvettes, 1 cm path length (3 mL reaction volume) at 20 °C. The stock solutions of the reactants were always added in the following order: PAANY, pyridine, imidazole and acetic anhydride **2**. Cuvettes were closed with Teflon caps and turned upside down to mix the solution. A fuel cycle was measured in continuous mode with 1000 measurements consisting of 11 runs each 3 min. An equilibration time of 2 min was applied for each measurement. Calculated size changes (%) are based on z-average diameters (nm). A DLS size calibration was performed to find the polymer concentration with minimal size fluctuation (see SI: DLS size calibration). The pH was always measured before and after the reaction.

1.5 Viscosity rheology measurements

For the blank experiments, 0.7 mL of the sample (PAANY (0.3 mM) in borate buffer 200 mM pH 8.0, borate buffer 200 mM pH 8.0 alone or borate buffer with 6 mM acetic anhydride fuel **2**) was directly positioned on the rheometer plate. For the PAANY acetylation experiment, the stock solutions of the reactants were added into a glass vial in the following order: PAANY, imidazole, pyridine and acetic anhydride **2**. The vial was turned upside down to mix the solution. Then, the 0.7 mL sample (0.3 mM PAANY, 0.75 mM imidazole, 0.3 mM pyridine, 6 mM acetic anhydride **2**) was positioned on the rheometer plate. Time sweep measurements were performed at fixed strain (γ = 0.05 %) and frequency (ω =6.28 rad/s = 1 Hz). Flow step measurements were performed after time sweep measurements on the same sample.

1.6 Kinetic modelling

A kinetic model for the esterification CRN was written in Matlab 2018b. The rate constant of imidazole catalysed ester hydrolysis was determined by varying the concentration of imidazole in the hydrolysis of *p*-nitrophenyl acetate **3** with UV-VIS, following the appearance of the hydrolysis product **1** at 400 nm. The other rate constants were taken from the literature or obtained from the Matlab model fitting (see SI: Kinetic model for fuel-driven esterification CRN).

2 UV-VIS spectroscopy

2.1 Calibration lines

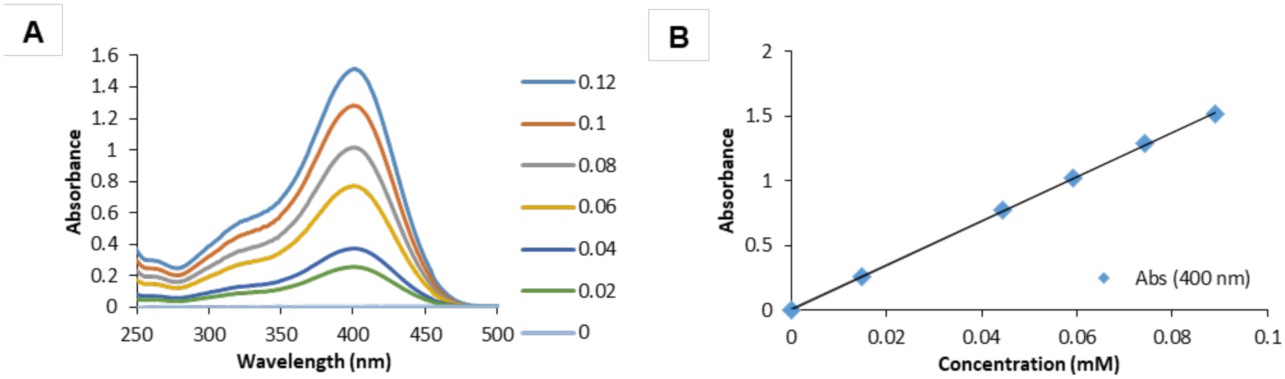

Figure S1: Extinction coefficient for p-nitrophenol (1) in MOPs buffer (100 mM, pH 7.5) at 400 nm: 16.74 $mM^{-1}cm^{-1}$. (A). UV-Vis absorbance spectra of *p*-nitrophenol (1) at different concentrations. (B). Absorbance at 400 nm of *p*-nitrophenol (1) at different concentrations.

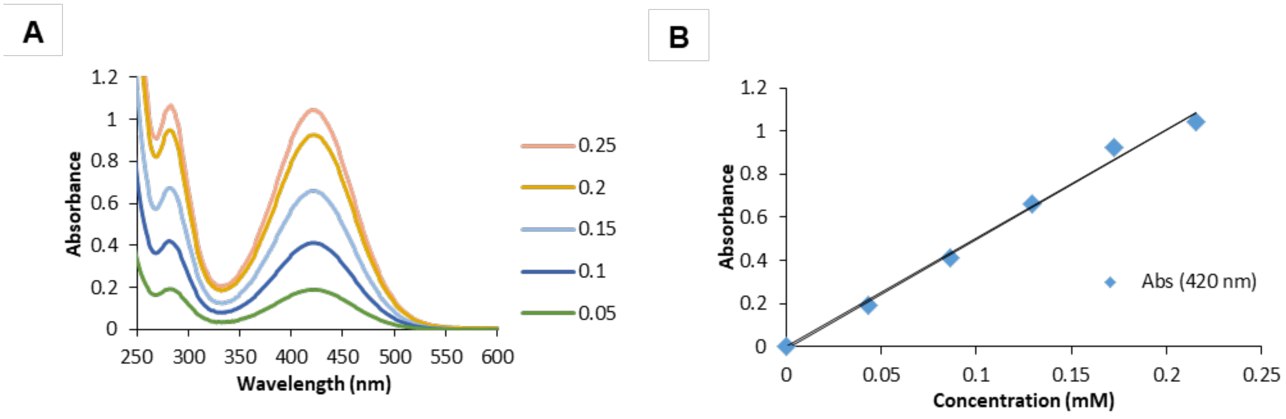

Figure S2: Extinction coefficient for 3-nitro-L-tyrosine (NY) in Borate buffer (200 mM, pH 8.0) at 420 nm: 5.03 $mM^{-1}cm^{-1}$. (A). UV-Vis absorbance spectra of 3-nitro-L-tyrosine (NY) at different concentrations. (B). Absorbance at 420 nm of 3-nitro-L-tyrosine (NY) at different concentrations.

2.2 Absorbance plots

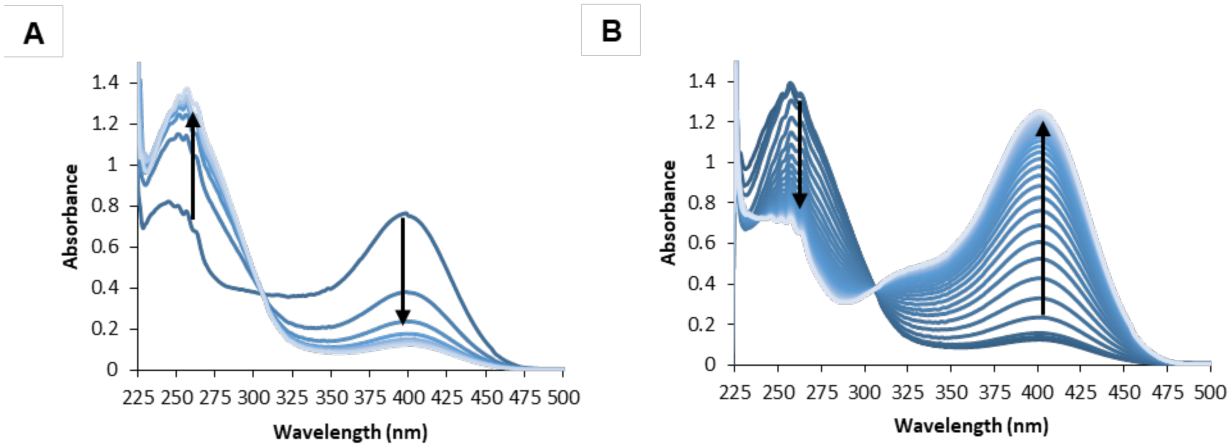

Figure S3: Absorbance vs wavelength for fuel-driven esterification network controlled by organocatalysts pyridine and imidazole in MOPS buffer (100 mM, pH 7.5) with 5% acetonitrile. Monitoring the conversion of *p*-nitrophenol(ate): 0.1 mM *p*-nitrophenol 1, 0.5 mM acetic anhydride 2 (stock in acetonitrile), 0.25 mM imidazole and 0.1 mM pyridine. (A) First 10 min of reaction, showing the decrease in 400 nm and increase for 270 nm – from dark to light blue (N.B. the first sample was measured after 10-30 s, hence part of the phenolate was already consumed). (B). From 10 min to 16.5 h, showing the increase in 400 nm and decrease for 270 nm - from dark to light blue. The isobestic point for this reaction is located at 305 nm. N.B Acetyl-imidazole disappearance is shown at 245 nm and acetyl-pyridinium at 272 nm [1].

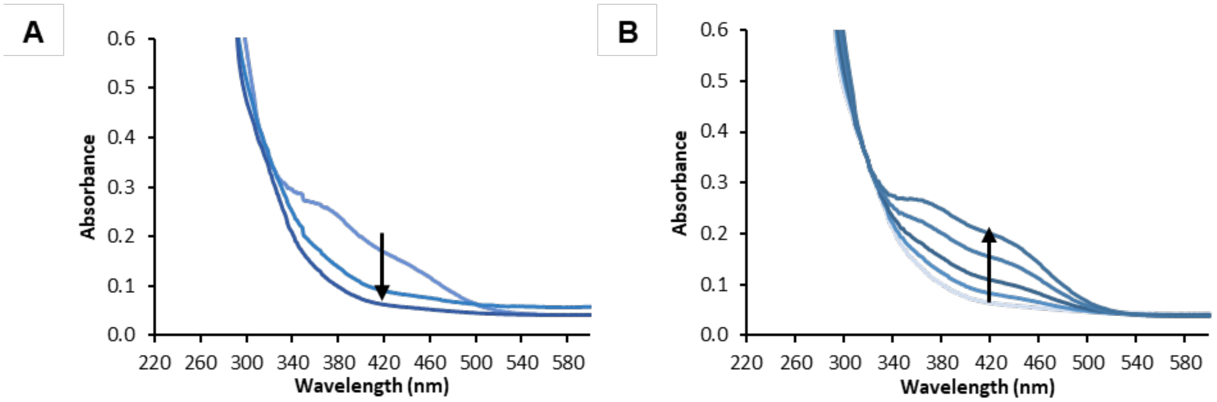

Figure S4: Absorbance vs wavelength for fuel-driven responsive polymer system controlled by organocatalysts pyridine and imidazole in borate buffer (200 mM, pH 8.0). Monitoring the conversion of PAANY by UV-VIS: 0.15 mM PAANY (0.12 mg/mL), 1.5 mM acetic anhydride 2, 0.375 mM imidazole and 0.15 mM pyridine. (A) First 5 min of reaction, showing the decrease in 420 nm (N.B. the first sample was measured after 10-30 s, hence part of the phenolate was already consumed). (B). From 5 min to 3 h, showing the increase in 420 nm.

3 Fuel cycle error analysis

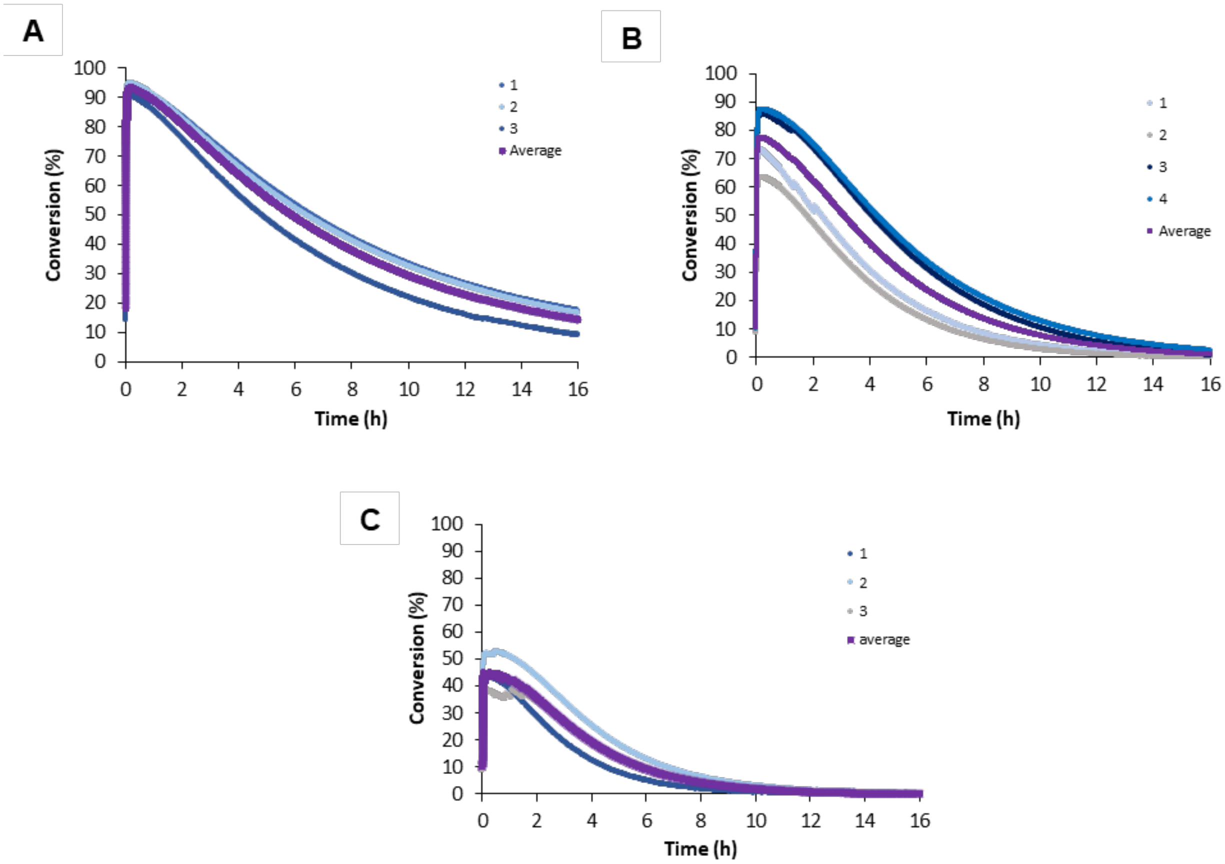

Figure S5: Error analysis of fuel-driven esterification network controlled by organocatalysts pyridine and imidazole in MOPS buffer (100 mM, pH 7.5) with 5% acetonitrile: (A). Monitoring the conversion of *p*-nitrophenol(ate): 0.1 mM *p*-nitrophenol 1, 0.5 mM acetic anhydride 2 (stock in acetonitrile), 0.1 mM pyridine and 0.1 mM imidazole, given in triplicate with average and the average standard deviation is 2.8% (n=3) . (B). Monitoring the conversion of *p*-nitrophenol(ate): 0.1 mM *p*-nitrophenol 1, 0.5 mM acetic anhydride 2 (stock in acetonitrile), 0.1 mM pyridine and 0.2 mM imidazole, given in quadruplicate with average and the average standard deviation is 10.5% (n=4). (C). Monitoring the conversion of *p*-nitrophenol(ate): 0.1 mM

p-nitrophenol 1, 0.5 mM acetic anhydride 2 (stock in acetonitrile), 0.1 mM pyridine and 0.3 mM imidazole, given in triplicate with average and the average standard deviation is 6.5% (n=3).**

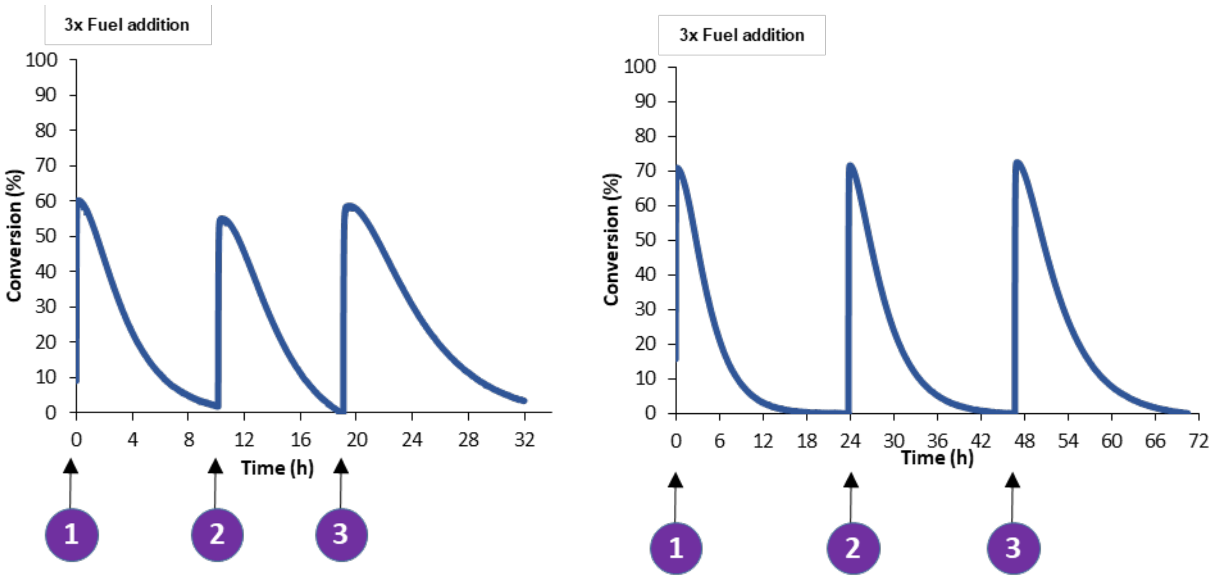

Figure S6: Three consecutive fuel cycles: 0.1 mM *p*-nitrophenol 1, 0.5 mM acetic anhydride 2 (stock in acetonitrile), 0.25 mM imidazole and 0.1 mM pyridine. The second and third cycles were initiated by addition of a new batch of fuel 2. The discrepancy between the first and second graph is most likely caused by a faster hydrolysis of the anhydride fuel 2 in the stock solution for graph 1 before addition to the reaction mixture. In Figure S5 the same discrepancies are observed, inherent to this system where the fuel consumption is very fast.

4 Blank reaction without catalysts

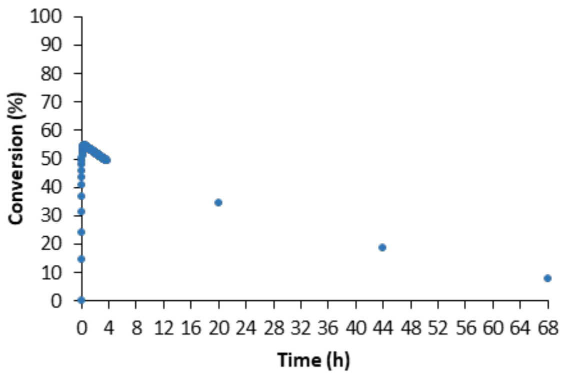

Figure S7: Blank reaction for fuel-driven esterification network in MOPS buffer (100 mM, pH 7.5) with 5% acetonitrile. Monitoring the conversion of *p*-nitrophenol(ate): 0.1 mM *p*-nitrophenol 1 with 0.5 mM acetic anhydride 2 (stock in acetonitrile).

5 HPLC-MS analysis

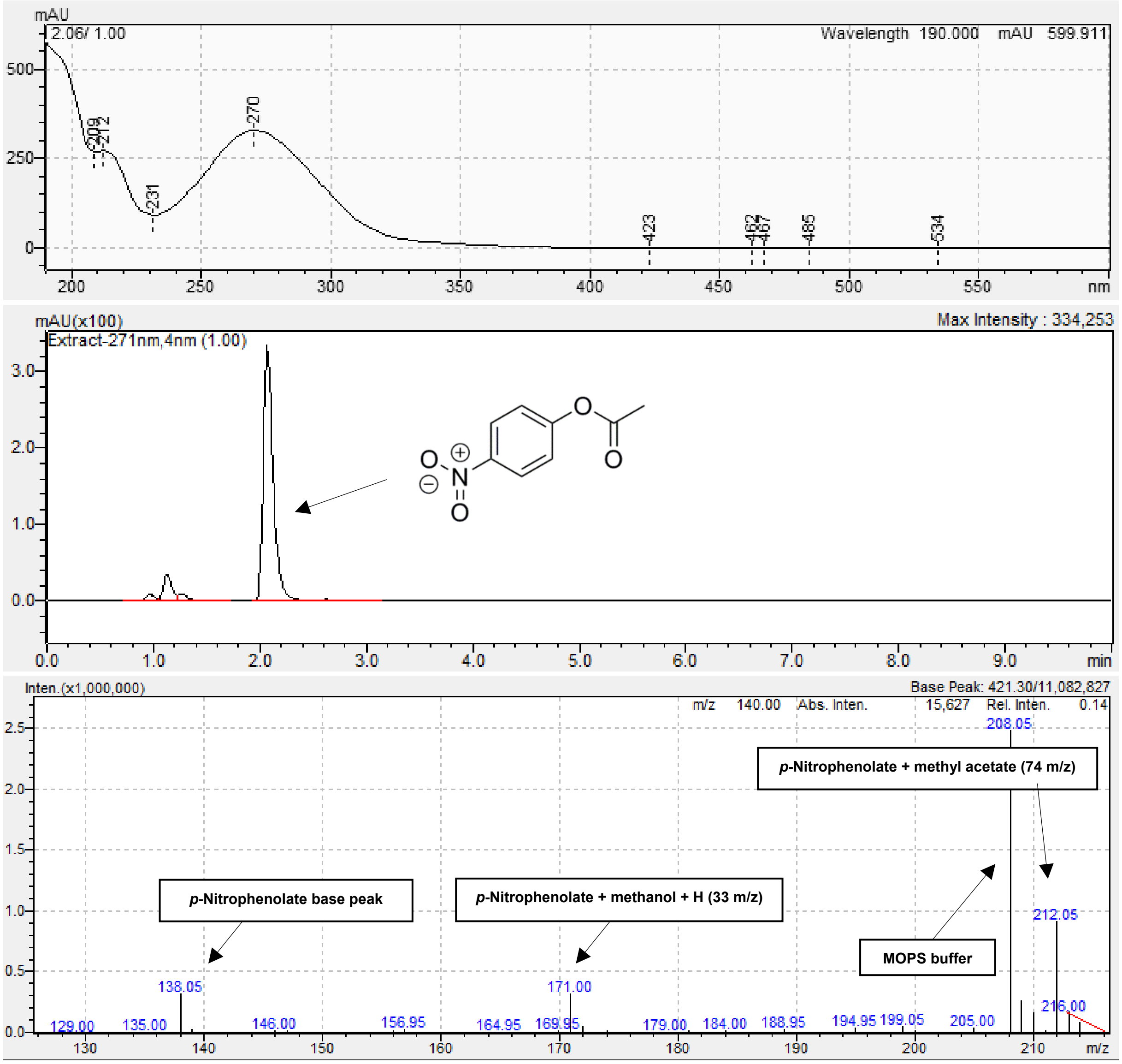

Figure S8: HPLC-MS: Fuel-driven esterification network controlled by organocatalysts pyridine and imidazole in MOPS buffer (100 mM, pH 7.5) with 5% acetonitrile. Conditions: 0.1 mM *p*-nitrophenol 1, 0.5 mM acetic anhydride 2 (stock in acetonitrile), 0.1 mM imidazole and 0.1 mM pyridine. Sample was measured 10 min after addition of all compounds. *p*-Nitrophenylacetate elutes at ~2.05 min, shows an absorbance maximum ~270 nm and 138 as m/z value (*p*-nitrophenyl esters give base peaks of the corresponding phenolate [2]).

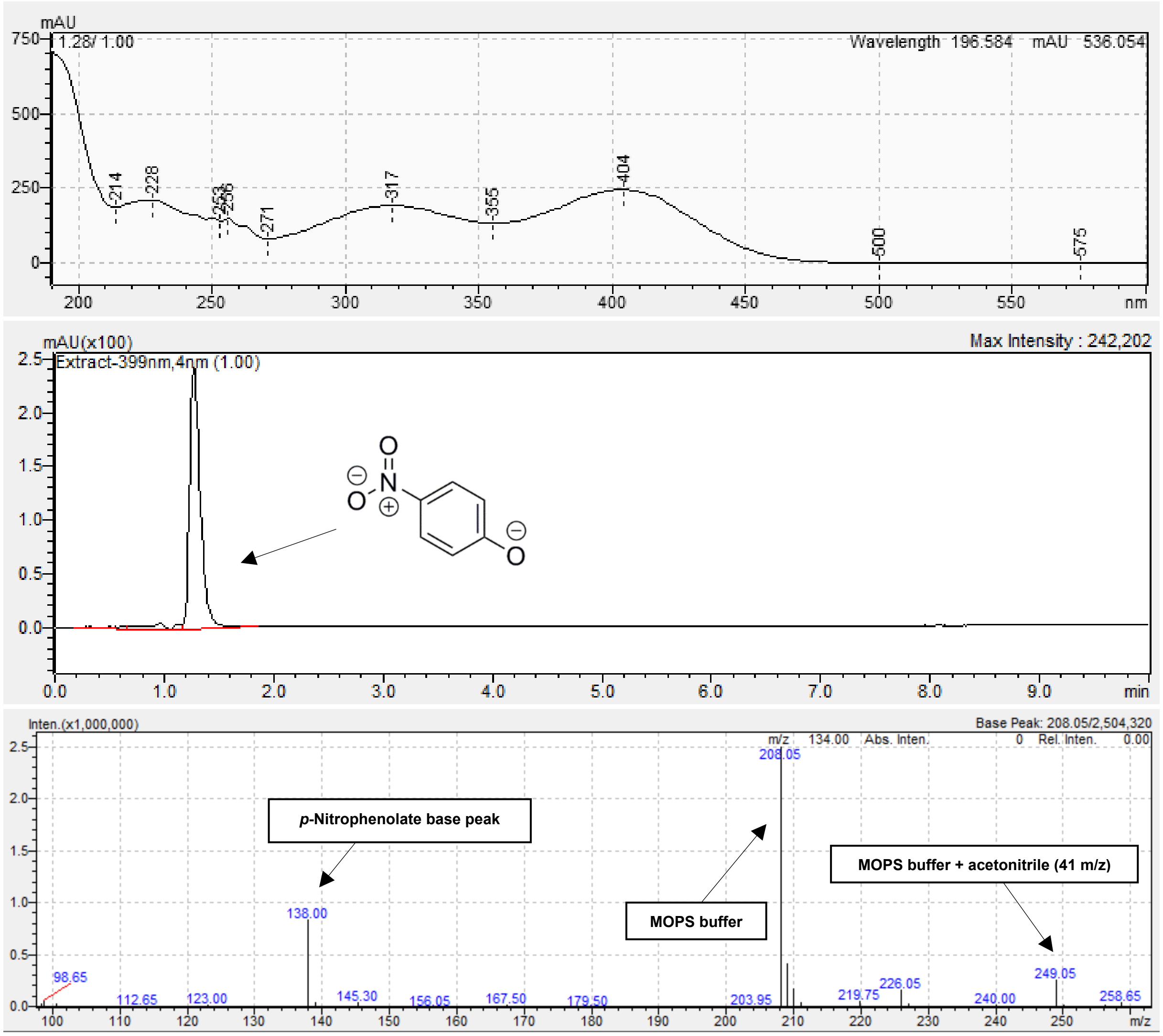

Figure S9: HPLC-MS: Fuel-driven esterification network controlled by organocatalysts pyridine and imidazole in MOPS buffer (100 mM, pH 7.5) with 5% acetonitrile. Conditions: 0.1 mM *p*-nitrophenol 1, 0.5 mM acetic anhydride 2 (stock in acetonitrile), 0.1 mM imidazole and 0.1 mM pyridine. Sample was measured 18h after addition of all compounds. *p*-Nitrophenol(ate) elutes at ~1.3 min, shows an absorbance maximum ~400 nm and 138 as m/z value.

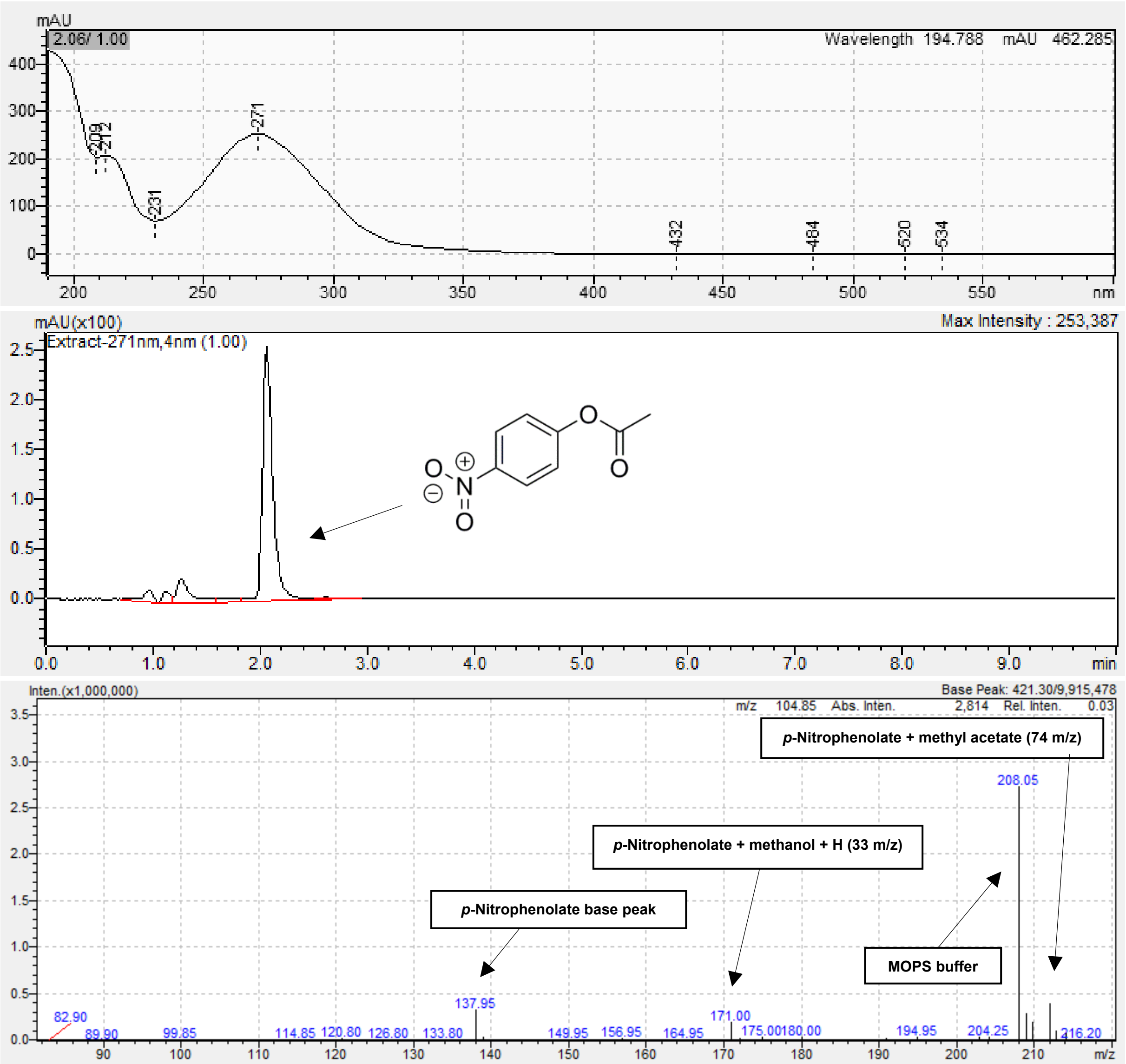

Figure S10: HPLC-MS: Blank reaction for fuel-driven esterification network in MOPS buffer (100 mM, pH 7.5) with 5% acetonitrile. Conditions: 0.1 mM *p*-nitrophenol 1 and 0.5 mM acetic anhydride 2 (stock in acetonitrile). Sample was measured 25 min after addition of all compounds. *p*-Nitrophenylacetate elutes at ~2.05 min, shows an absorbance maximum ~270 nm and 138 as m/z value (*p*-nitrophenyl esters give base peaks of the corresponding phenolate [2]).

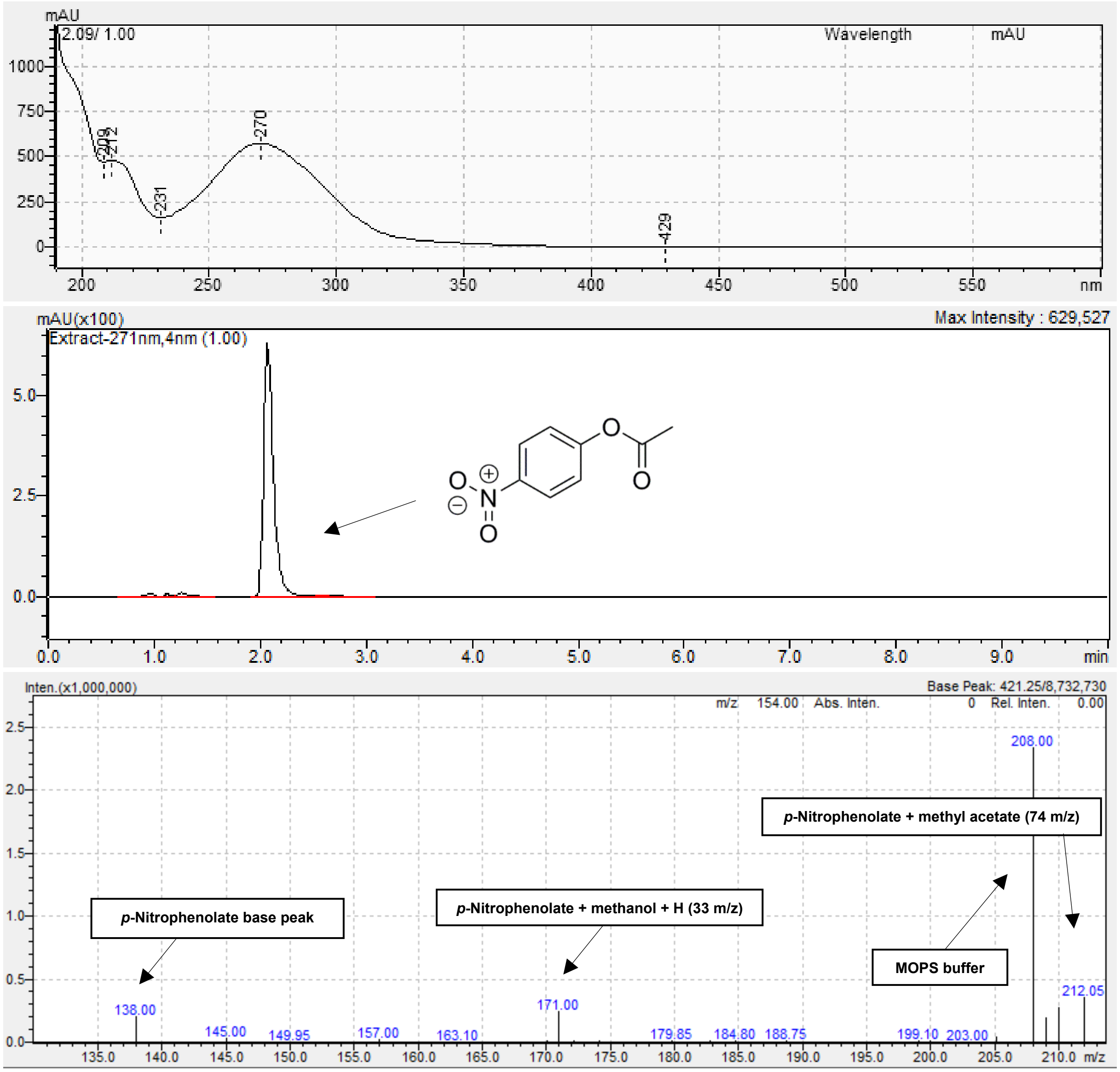

Figure S11: HPLC-MS: *p*-Nitrophenylacetate reference compound in MOPS buffer (100 mM, pH 7.5) with 5% acetonitrile elutes at ~2.05 min, shows an absorbance maximum ~270 nm and 138 as m/z value (*p*-nitrophenyl esters give base peaks of the corresponding phenolate [2]).

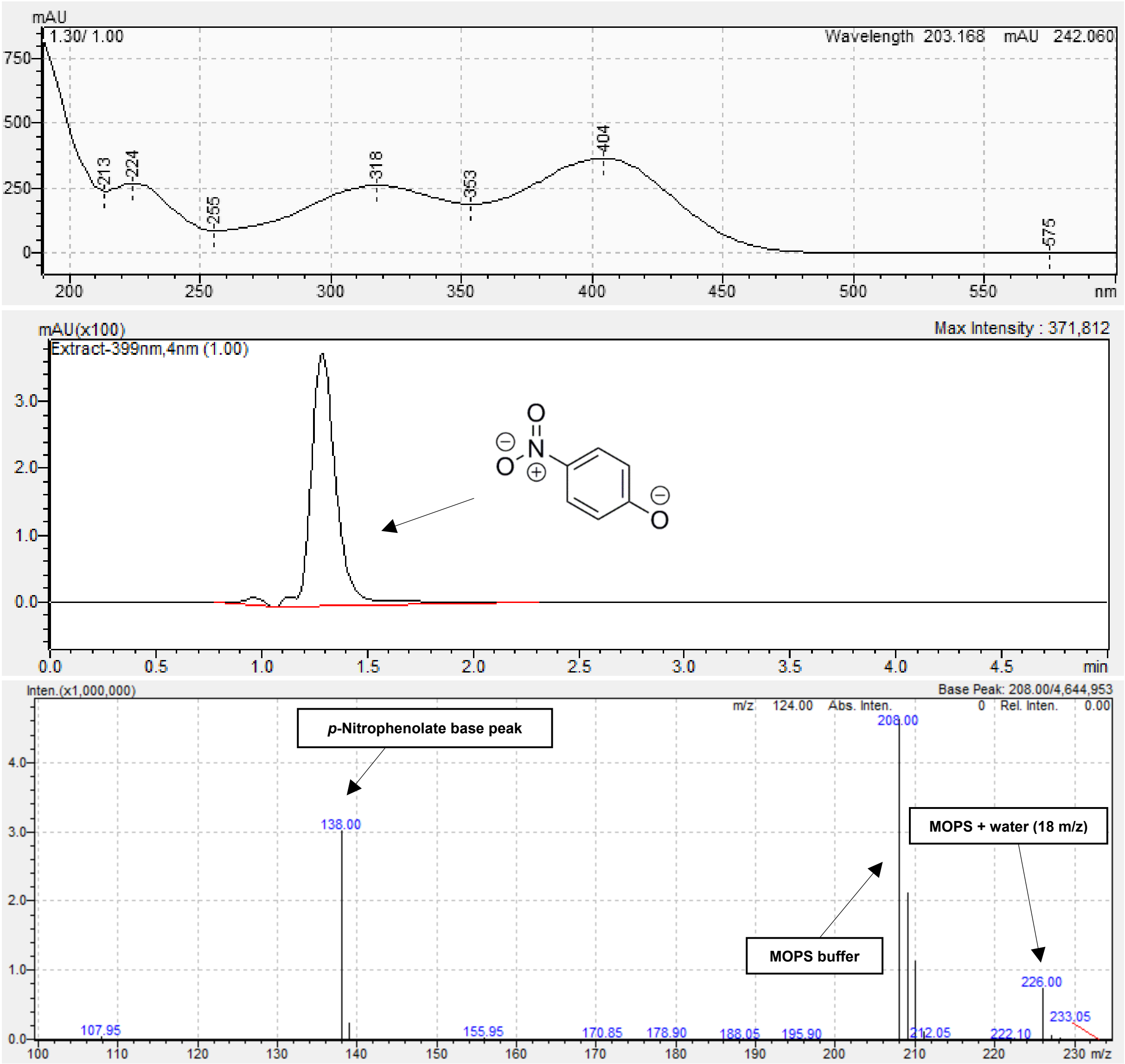

Figure S12: HPLC-MS: *p*-Nitrophenol(ate) reference compound in MOPS buffer (100 mM, pH 7.5) elutes at ~1.3 min, shows an absorbance maximum ~400 nm and 138 as m/z value.

6 Monitoring esterification network by color progress

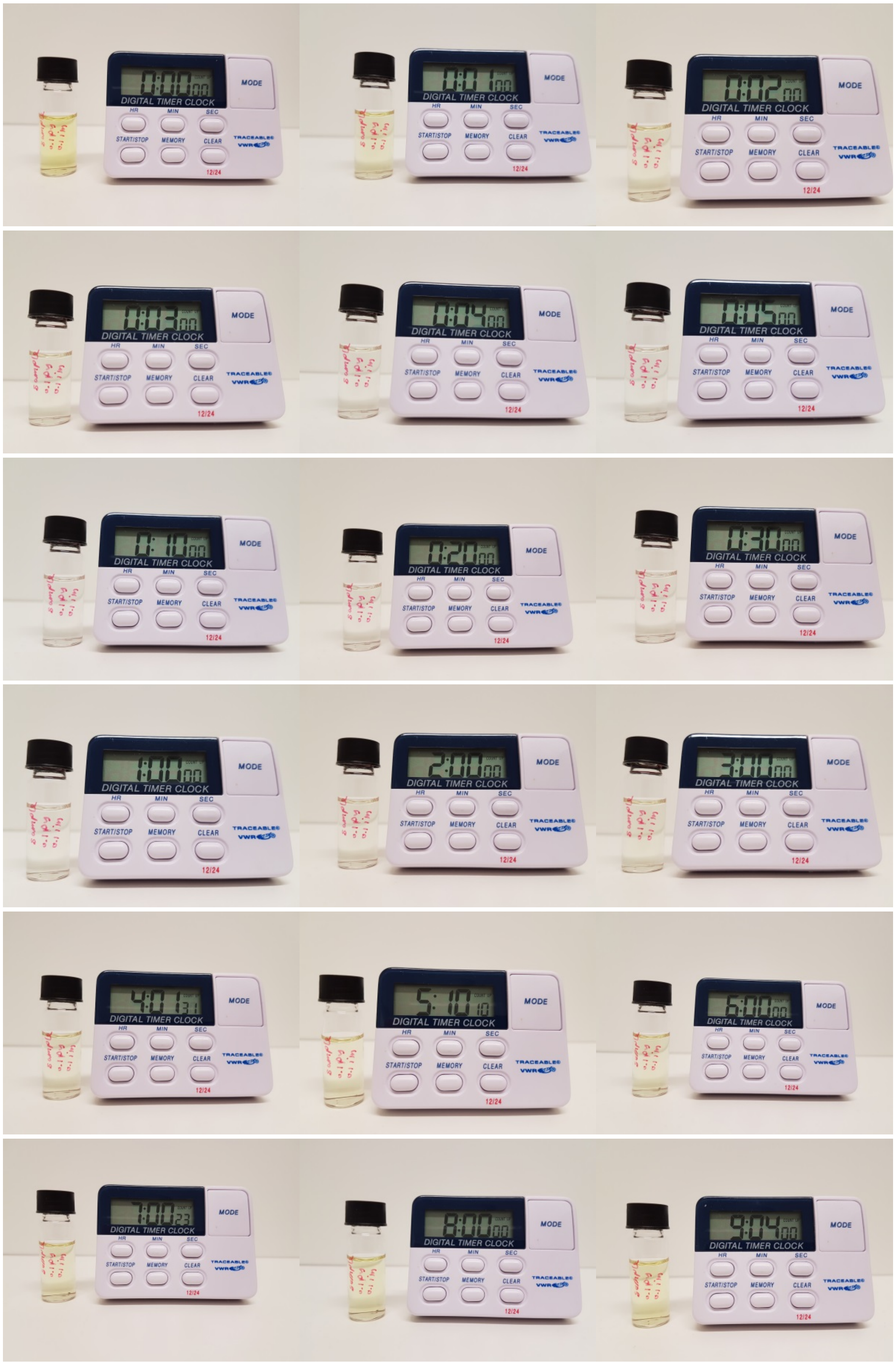

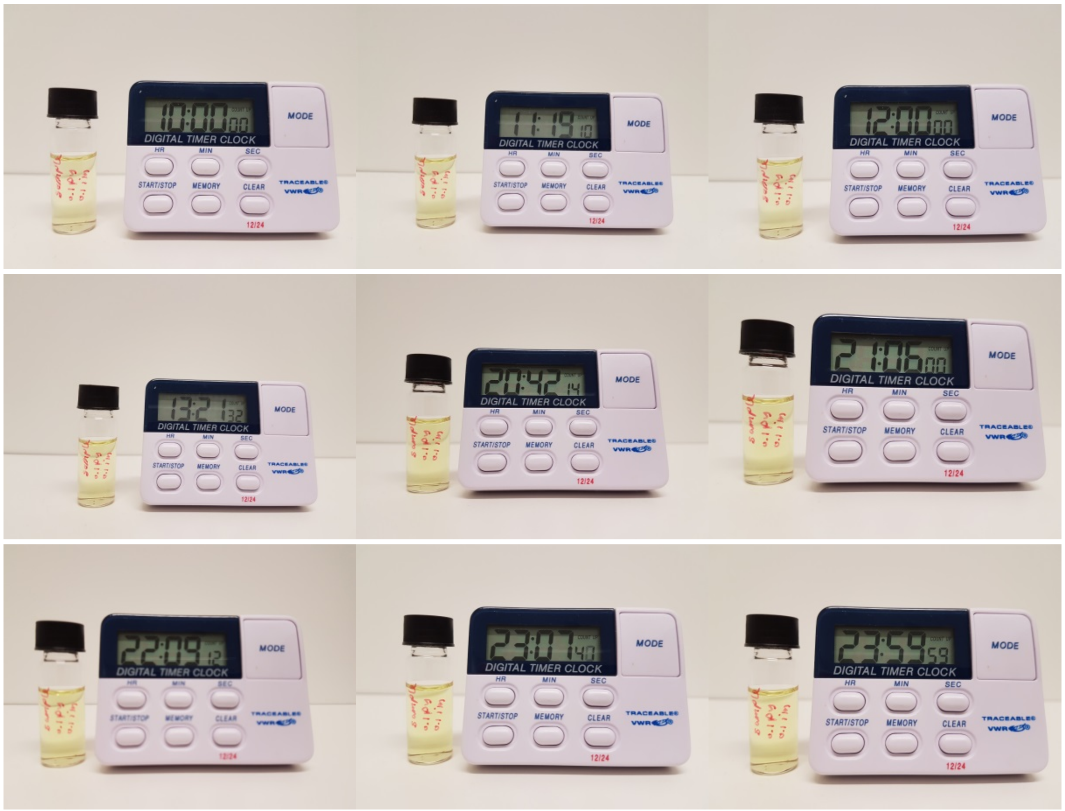

Figure S13: Fuel-driven esterification network controlled by organocatalysts pyridine and imidazole in MOPS buffer (100 mM, pH 7.5) with 5% acetonitrile, monitoring reaction by colour progress; yellow to transparent and back to yellow again (Conditions: 0.1 mM *p*-nitrophenol 1, 0.5 mM acetic anhydride 2 (stock in acetonitrile), 0.1 mM imidazole and 0.1 mM pyridine).

7 pH monitoring of fuel-driven esterification network

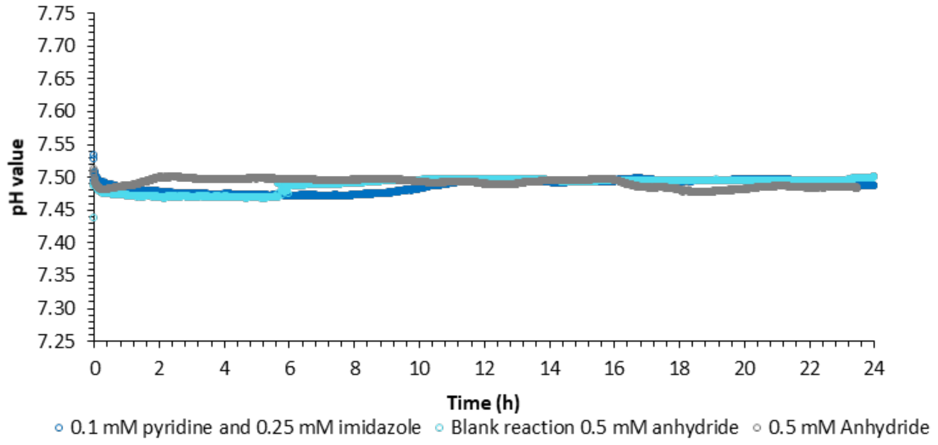

Figure S14: pH monitoring of reaction with organocatalysts, blank reaction and anhydride stock in MOPS buffer pH 7.5 100 mM.

8 Different acyl donors

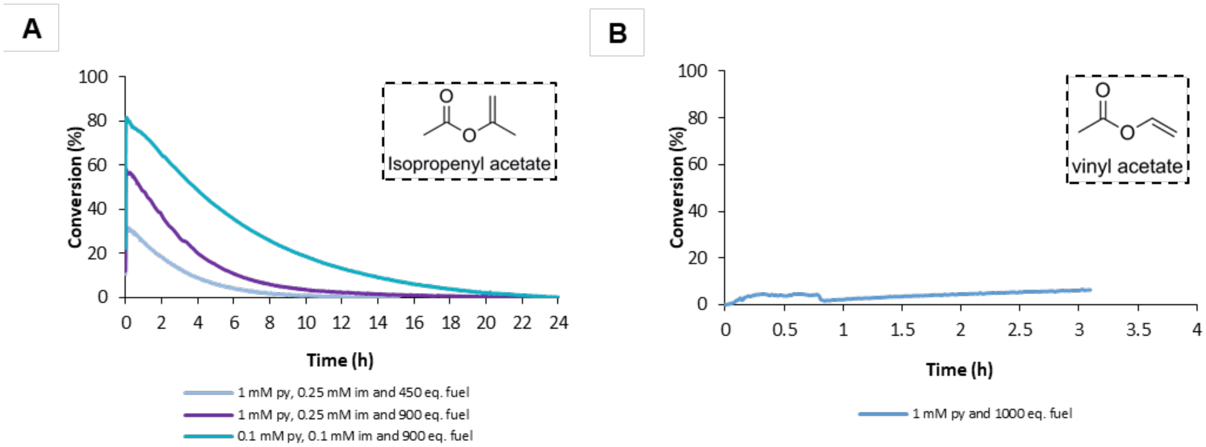

Figure S15: Fuel-driven esterification network with varying concentration of organocatalysts, monitoring the conversion of *p*-nitrophenol(ate): (A). 450-900 molar eq. isopropenyl acetate. (B). 1000 molar eq. vinyl acetate. 0.1 mM *p*-nitrophenol 1 in MOPS buffer (100 mM, pH 7.5), pyridine (0.1-1 mM) and imidazole (0.1-0.25 mM).

Table S1: Overview of tested acyl donors as fuels for esterification of *p*-nitrophenol(ate) (1).

Fuel	Structure	Waste product(s)	UV-VIS	Remarks
Acetic anhydride	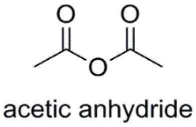	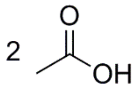	5 molar eq. of fuel to drive the esterification	Acetic anhydride completely hydrolysed after 30 minutes. Then only backward reactions remain.
Isopropenyl acetate	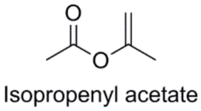	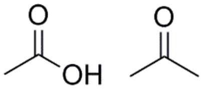	450-900 molar eq. of fuel (45-90 mM) needed to drive the reaction	- Heterogeneous mixture (fuel hardly dissolves in water and creates little bubbles). - Due to high concentration of fuel pH drops to 7.46 at the end of the reaction.
Vinyl acetate	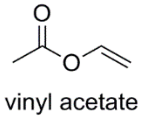	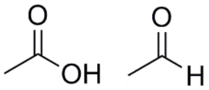	1000 molar eq. of fuel (100 mM) not sufficient	

9 Kinetic model for fuel-driven esterification CRN

In this section the development of the numerical model for the reaction kinetics of the esterification CRN written in Matlab 2018b is discussed. First we deal with the kinetics of the blank reaction (uncatalysed), then the pyridine catalysis, followed by imidazole catalysis and eventually all individual reaction schemes are combined to model the entire fuel cycle (Scheme S1) with varying catalysts and fuel concentrations. In all cases, first an overview of the reaction pathway is given, followed by the rate equations (system of ODEs) and the experimental data fitting, showing the concentration profiles of the different species over time for the modelled and experimental data. We end with a note on how the model was optimized for the various experimental conditions.

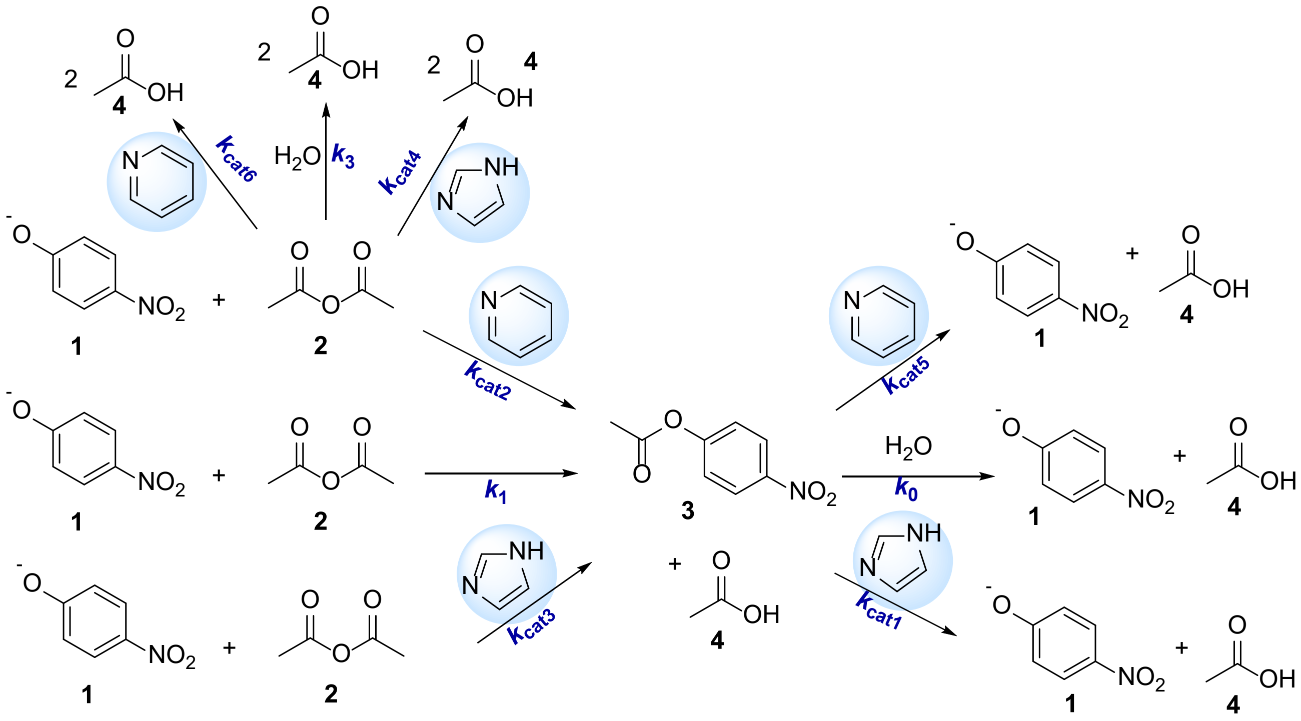

Scheme S1: Full reaction pathway overview, including organocatalysed and blank (uncatalysed) reactions. Both pyridine and imidazole catalyse the ester formation, hydrolysis and anhydride hydrolysis.

9.1 Blank reaction

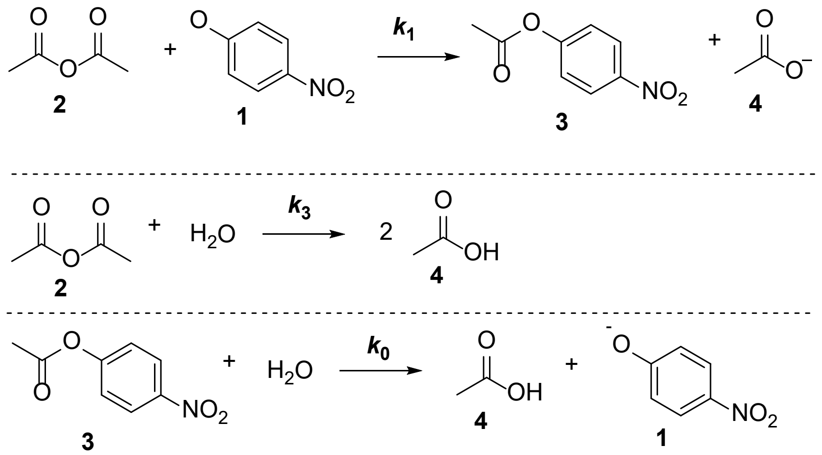

Rate equations

When only the blank reaction takes place, the following equations apply:

Formation and degradation of the ester (**3**)

$$\frac{d[\mathbf{3}]}{dt} = k_1[\mathbf{1}][\mathbf{2}] - k_0[\mathbf{3}]$$

Formation of the acid (**4**)

$$\frac{d[\mathbf{4}]}{dt} = k_1[\mathbf{1}][\mathbf{2}] + 2k_3[\mathbf{2}] + k_0[\mathbf{3}]$$

Formation and degradation of the phenolate (**1**)

$$-\frac{d[\mathbf{1}]}{dt} = k_1[\mathbf{1}][\mathbf{2}] - k_0[\mathbf{3}]$$

Degradation of the anhydride (**2**)

$$-\frac{d[\mathbf{2}]}{dt} = k_1[\mathbf{1}][\mathbf{2}] + k_3[\mathbf{2}]$$

Experimental data fitting

Using k_0 0.0001 min^{-1} (blank hydrolysis – determined experimentally), the literature value for k_3 0.1575 min^{-1} for the hydrolysis of acetic anhydride **2** [3] and k_1 0.25 $mM^{-1}min^{-1}$ as initial guesses. k_1 was determined by fitting the data of the blank reaction, giving k_1 0.3750 $mM^{-1}min^{-1}$, k_3 0.2219 min^{-1} and k_0 0.0001 min^{-1}.

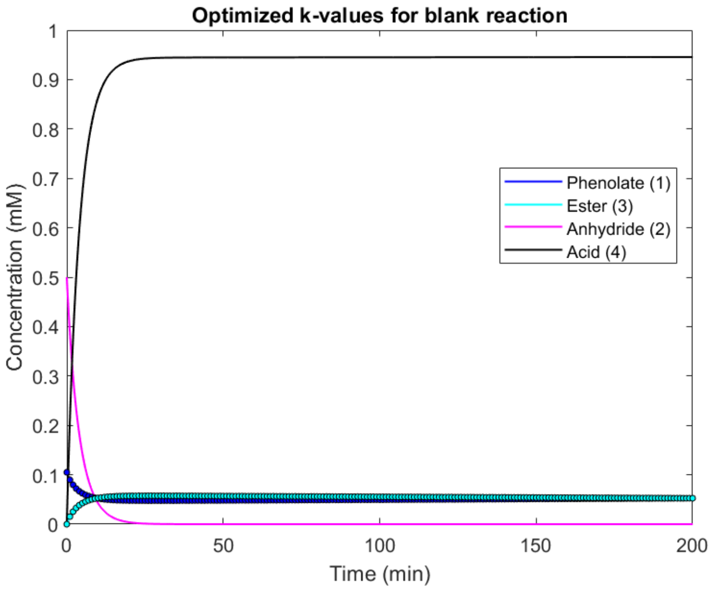

Figure S16: Concentration profiles of different species (phenolate 1, ester 3, acetic anhydride 2 and acetic acid 4) over time for the blank reaction (uncatalysed): experimental data (dots) and model (lines) with new *k*-values k_1 0.375 $mM^{-1}min^{-1}$, k_3 0.2219 min^{-1} and k_0 0.0001 min^{-1}.

9.2 Pyridine catalysis and blank reaction

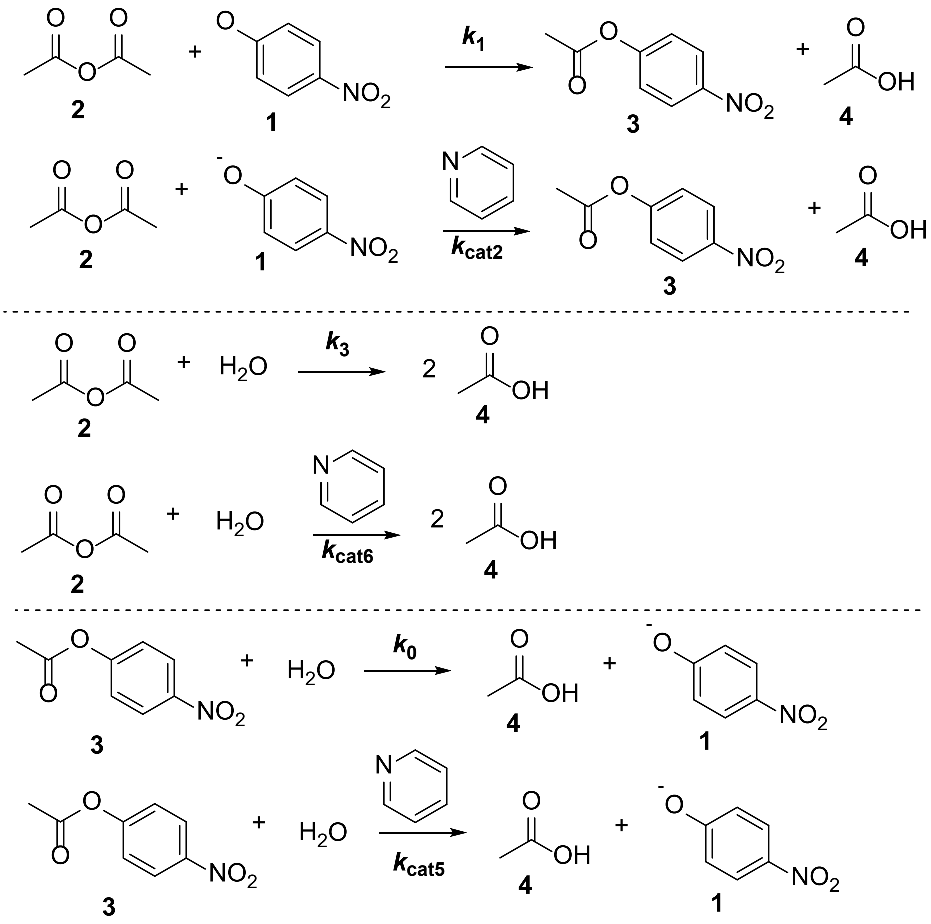

Rate equations

Next, when only the blank reaction takes place and the pyridine catalysis, the following equations apply:

Formation and degradation of the ester (**3**)

$$\frac{d[\mathbf{3}]}{dt} = k_1[\mathbf{1}][\mathbf{2}] + k_{cat2}[\boldsymbol{Py}][\mathbf{1}][\mathbf{2}] - k_0[\mathbf{3}] - k_{cat5}[\boldsymbol{Py}][\mathbf{3}]$$

Formation of the acid (**4**)

$$\frac{d[\mathbf{4}]}{dt} = k_1[\mathbf{1}][\mathbf{2}] + k_{cat2}[\boldsymbol{Py}][\mathbf{1}][\mathbf{2}] + 2k_3[\mathbf{2}] + k_0[\mathbf{3}] + k_{cat5}[\boldsymbol{Py}][\mathbf{3}] + 2\,k_{cat6}[\boldsymbol{Py}][\mathbf{2}]$$

Formation and degradation of the phenolate (**1**)

$$-\frac{d[\mathbf{1}]}{dt} = k_1[\mathbf{1}][\mathbf{2}] + k_{cat2}[\boldsymbol{Py}][\mathbf{1}][\mathbf{2}] - k_0[\mathbf{3}] - k_{cat5}[\boldsymbol{Py}][\mathbf{3}]$$

Degradation of the anhydride (**2**)

$$-\frac{d[\mathbf{2}]}{dt} = k_1[\mathbf{1}][\mathbf{2}] + k_{cat2}[\boldsymbol{Py}][\mathbf{2}] + k_3[\mathbf{2}] + \ k_{cat6}[\boldsymbol{Py}][\mathbf{2}]$$

Experimental data fitting

The previous k-values from the blank reaction were again used to determine k_{cat2} 35.0 mM^{-2}min^{-1}, k_{cat5} 0.002 mM^{-1}min^{-1} and k_{cat6} 1.525 mM^{-1}min^{-1}.

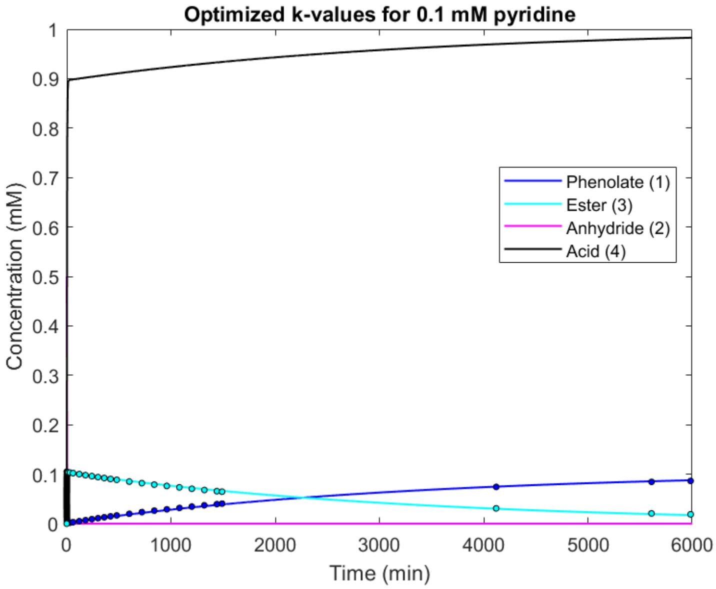

Figure S17: Concentration profiles of different species (phenolate 1, ester 3, acetic anhydride 2 and acetic acid 4) over time for the pyridine (0.1 mM) catalysed ester formation: Experimental data (dots) and model (line) with new *k*-values k_{cat2} 35.0 mM^{-2}min^{-1}, k_{cat5} 0.002 mM^{-1}min^{-1} and k_{cat6} 1.525 mM^{-1}min^{-1}.

9.3 Imidazole catalysis and blank reaction

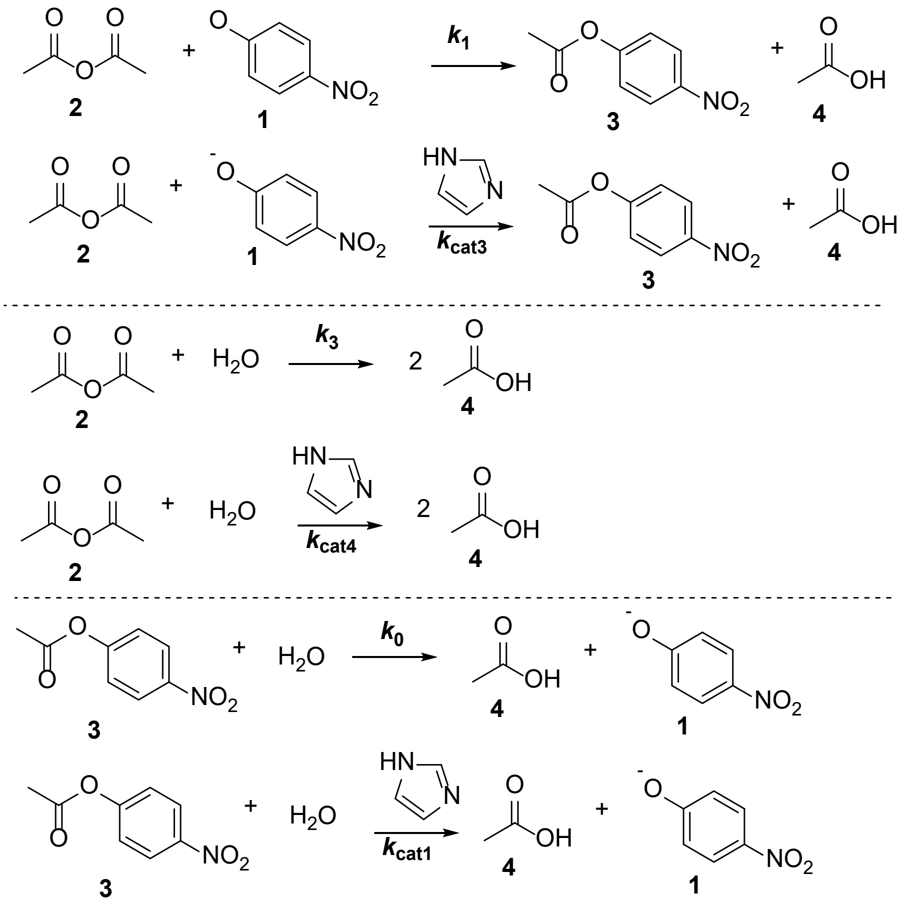

Rate equations

When only the blank reaction takes place and the imidazole catalysis, the following equations apply:

Formation and degradation of the ester (**3**)

$$\frac{d[\mathbf{3}]}{dt} = k_1[\mathbf{1}][\mathbf{2}] + k_{cat3}[\boldsymbol{Im}][\mathbf{1}][\mathbf{2}] - k_0[\mathbf{3}] - k_{cat1}[\boldsymbol{Im}][\mathbf{3}]$$

Formation of the acid (**4**)

$$\frac{d[\mathbf{4}]}{dt} = k_1[\mathbf{1}][\mathbf{2}] + k_{cat3}[\boldsymbol{Im}][\mathbf{1}][\mathbf{2}] + 2k_3[\mathbf{2}] + k_0[\mathbf{3}] + k_{cat1}[\boldsymbol{Im}][\mathbf{3}] + 2\,k_{cat4}[\boldsymbol{Im}][\mathbf{2}]$$

Formation and degradation of the phenolate (**1**)

$$-\frac{d[\mathbf{1}]}{dt} = k_1[\mathbf{1}][\mathbf{2}] + k_{cat3}[\boldsymbol{Im}][\mathbf{1}][\mathbf{2}] - k_0[\mathbf{3}] - k_{cat1}[\boldsymbol{Im}][\mathbf{3}]$$

Degradation of the anhydride (**2**)

$$-\frac{d[\mathbf{2}]}{dt} = k_1[\mathbf{1}][\mathbf{2}] + k_{cat3}[\boldsymbol{Im}][\mathbf{1}][\mathbf{2}] + k_3[\mathbf{2}] + k_{cat4}[\boldsymbol{Im}][\mathbf{2}]$$

Experimental data fitting

k_{cat1} and k_0 were determined experimentally (k_{cat1} 0.475 $M^{-1}s^{-1}$ and k_0 -4.0·10^{-5} s^{-1}) (Table S2 comparison with literature). Yet, the k-values were optimized with Matlab to fit the experimental data better (N.B. data for 5 mM imidazole were used as experimental values). Lower and upper bounds for the k-values were opposed with the fmincon constrained optimization function using a least squared cost function. The new k-values are: k_{cat} 0.0254 $mM^{-1}min^{-1}$ (0.423 $M^{-1}s^{-1}$) and k_0 0.0001 min^{-1} (1.67·10^{-6} s^{-1}) .

Table S2: Rate constants comparison against literature values.

	**This work**	**Bruice[4]**	**Bruice[4]**	**Lombardo[5]**
**k_{cat1} ($M^{-1}s^{-1}$)**	0.475 ± 0.0034	0.211 ± 0.0035	0.328 ± 0.0152	0.130
**k_0 (s^{-1})**	-4.0·10^{-5}	9.0 ·10^{-6}	7.33 ·10^{-5}	4.35 ·10^{-5}
**Buffer**	MOPS pH 7.5 100 mM	Phosphate pH 7.9-8.0 5.4 mM	Phosphate pH 7.9-8.0 200 mM	Imidazole in 0.1 M KCl
**Temperature**	21 °C (294 K)	25 °C (298 K)	30 °C (298 K)	25 °C (298 K)
**Method**	Abs 400 nm	Abs 400 nm	Abs 400 nm	Abs 400 nm
**Remarks**	Corrected for imidazole free base species	Corrected for imidazole free base species	Corrected for imidazole free base species	Pure imidazole buffers used

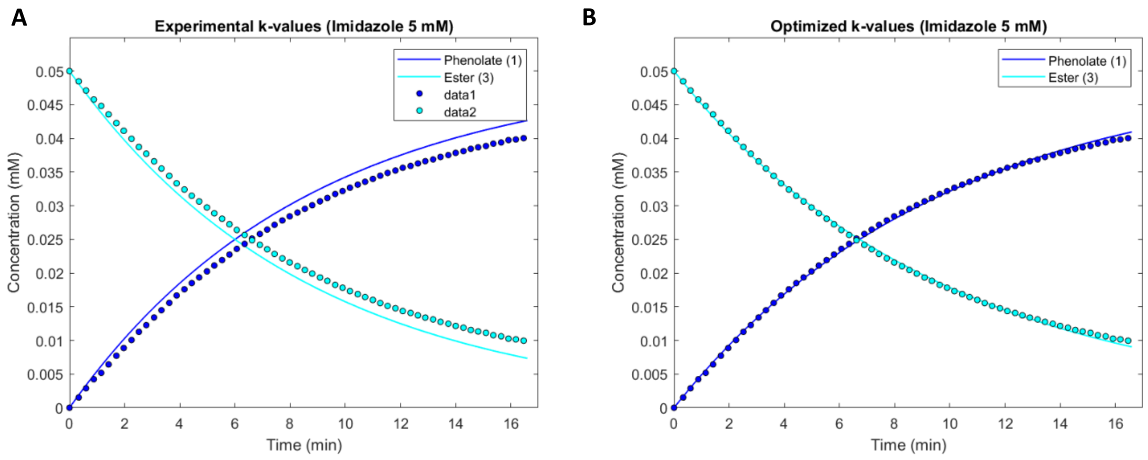

Figure S18: Optimization of k-values with fmincon: (A). Experimental data (dots) and model (line) with experimentally obtained k-values (k_{cat1} 0.475 $M^{-1}s^{-1}$ and k_0 -4.0·10^{-5} s^{-1}). (B). Experimental data (dots) and model (line) with new k-values k_{cat1} 0.0254 $mM^{-1}min^{-1}$ and k_0 0.00010 min^{-1}.

Then, the previous k-values were again used to determine k_{cat3} 0.124 $mM^{-2}min^{-1}$ and k_{cat4} 0.0565 $mM^{-1}min^{-1}$.

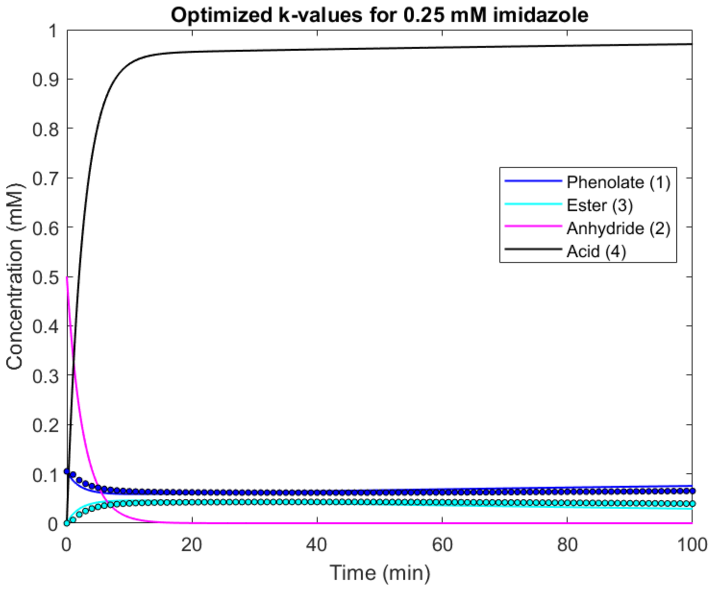

Figure S19: Concentration profiles of different species (phenolate 1, ester 3, acetic anhydride 2 and acetic acid 4) over time for the imidazole (0.25 mM) catalysed ester formation: Experimental data (dots) and model (line) with new k-values k_{cat3} 0.124 $mM^{-2}min^{-1}$ and k_{cat4} 0.0565 $mM^{-1}min^{-1}$.

9.4 Pyridine and imidazole catalysed reaction cycle

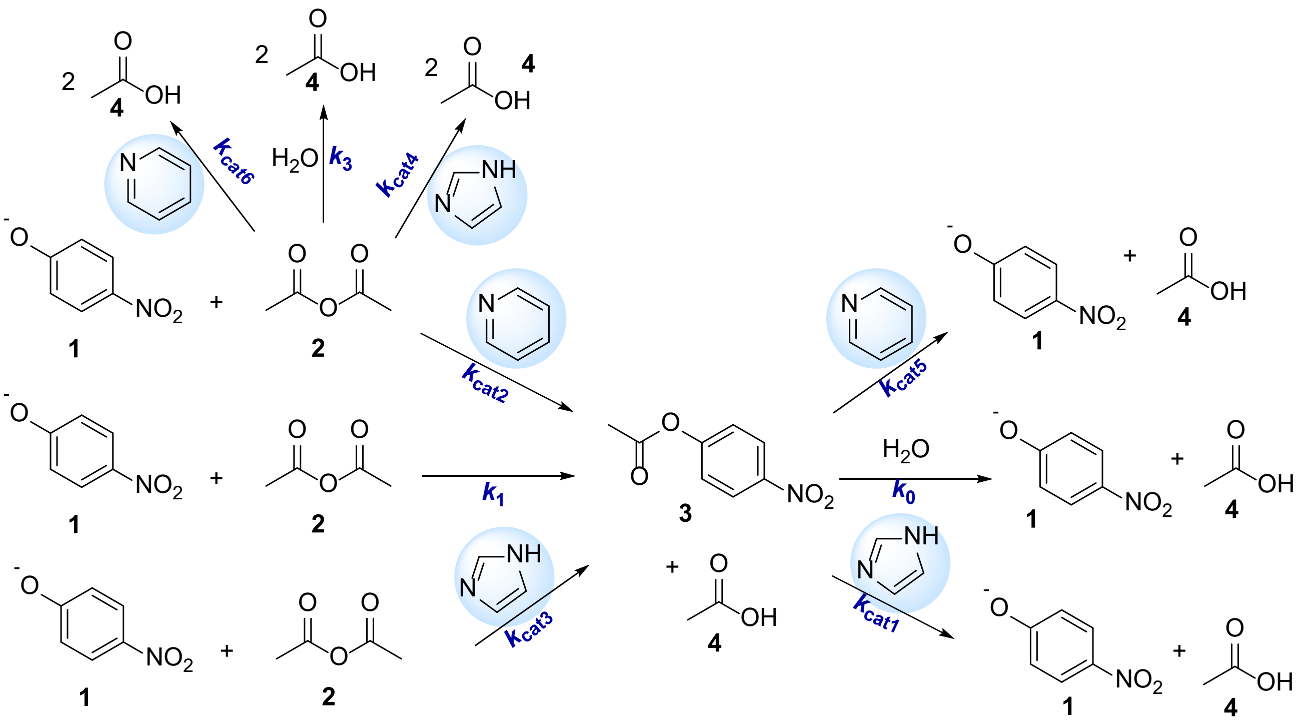

Rate equations

When the blank reaction, pyridine and imidazole catalysis take place the following equations apply:

Formation and degradation of the ester (**3**)

$$\frac{d[\mathbf{3}]}{dt} = k_1[\mathbf{1}][\mathbf{2}] + k_{cat2}[\boldsymbol{Py}][\mathbf{1}][\mathbf{2}] - k_0[\mathbf{3}] - k_{cat5}[\boldsymbol{Py}][\mathbf{3}] + k_{cat3}[\boldsymbol{Im}][\mathbf{1}][\mathbf{2}] - k_{cat1}[\boldsymbol{Im}][\mathbf{3}]$$

Formation of the acid (**4**)

$$\frac{d[\mathbf{4}]}{dt} = k_1[\mathbf{1}][\mathbf{2}] + k_{cat2}[\boldsymbol{Py}][\mathbf{1}][\mathbf{2}] + 2k_3[\mathbf{2}] + k_0[\mathbf{3}] + k_{cat5}[\boldsymbol{Py}][\mathbf{3}] + 2\,k_{cat6}[\boldsymbol{Py}][\mathbf{2}] + k_{cat1}[\boldsymbol{Im}][\mathbf{3}] + k_{cat3}[\boldsymbol{Im}][\mathbf{1}][\mathbf{2}] + 2\,k_{cat4}[\boldsymbol{Im}][\mathbf{2}]$$

Formation and degradation of the phenolate (**1**)

$$-\frac{d[\mathbf{1}]}{dt} = k_1[\mathbf{1}][\mathbf{2}] + k_{cat2}[\boldsymbol{Py}][\mathbf{1}][\mathbf{2}] - k_0[\mathbf{3}] - k_{cat5}[\boldsymbol{Py}][\mathbf{3}] + k_{cat3}[\boldsymbol{Im}][\mathbf{1}][\mathbf{2}] - k_{cat1}[\boldsymbol{Im}][\mathbf{3}]$$

Degradation of the anhydride (**2**)

$$-\frac{d[\mathbf{2}]}{dt} = k_1[\mathbf{1}][\mathbf{2}] + k_{cat2}[\boldsymbol{Py}][\mathbf{1}][\mathbf{2}] + k_3[\mathbf{2}] + k_{cat6}[\boldsymbol{Py}][\mathbf{2}] + k_{cat3}[\boldsymbol{Im}][\mathbf{1}][\mathbf{2}] + k_{cat4}[\boldsymbol{Im}][\mathbf{2}]$$

Experimental data fitting

All the simulated and experimentally determined k-values were again used to fit the esterification cycle with different pyridine, imidazole and acetic anhydride concentrations. In the next figures the fits are provided together with the specific k-values (optimized if needed).

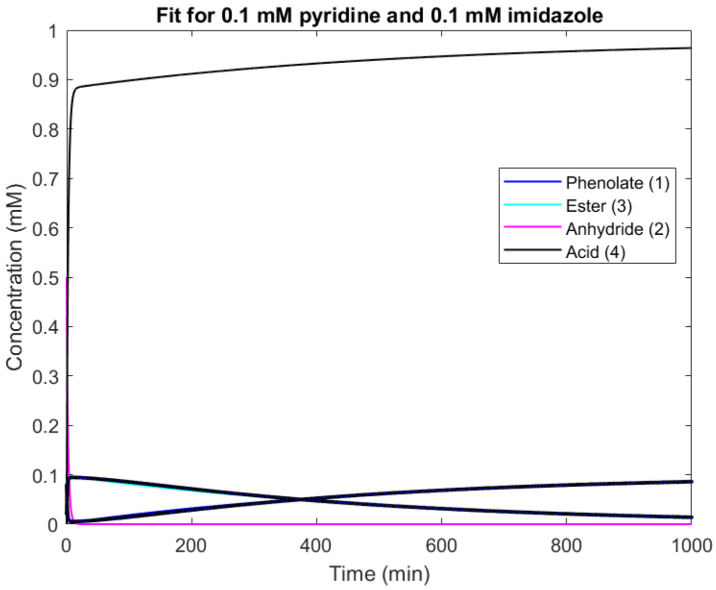

***k*-values**	
k_0	0.0002 min^{-1}
k_1	0.485 $mM^{-1}min^{-1}$
k_3	0.118 min^{-1}
k_{cat1}	0.0147 $mM^{-1}min^{-1}$
k_{cat2}	26.2377 $mM^{-2}min^{-1}$
k_{cat3}	0.0873 $mM^{-2}min^{-1}$
k_{cat4}	0.2258 $mM^{-1}min^{-1}$
k_{cat5}	0.0046 $mM^{-1}min^{-1}$
k_{cat6}	2.266 $mM^{-1}min^{-1}$

Figure S20: Concentration profiles of different species (phenolate 1, ester 3, acetic anhydride 2 and acetic acid 4) over time for the ester formation with pyridine (0.1 mM) and imidazole (0.1 mM): Eperimental data (black dots) and model (lines).

Increasing imidazole concentration (0.1 mM → 0.2 mM→ 0.25 mM → 0.3 mM → 0.5 mM → 1 mM)

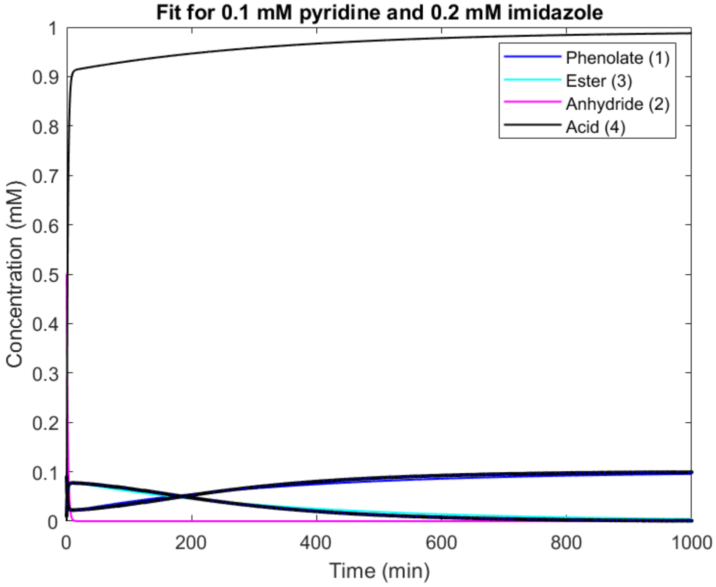

***k*-values**	
k_0	0.0001 min^{-1}
k_1	0.1993 $mM^{-1}min^{-1}$
k_3	0.1257 min^{-1}
k_{cat1}	0.0151 $mM^{-1}min^{-1}$
k_{cat2}	16.5306 $mM^{-2}min^{-1}$
k_{cat3}	0.0376 $mM^{-2}min^{-1}$
k_{cat4}	0.0565 $mM^{-1}min^{-1}$
k_{cat5}	0.0046 $mM^{-1}min^{-1}$
k_{cat6}	3.36620 $mM^{-1}min^{-1}$

Figure S21: Concentration profiles of different species (phenolate 1, ester 3, acetic anhydride 2 and acetic acid 4) over time for the ester formation with pyridine (0.1 mM) and imidazole (0.2 mM): Experimental data (black dots) and model (lines).

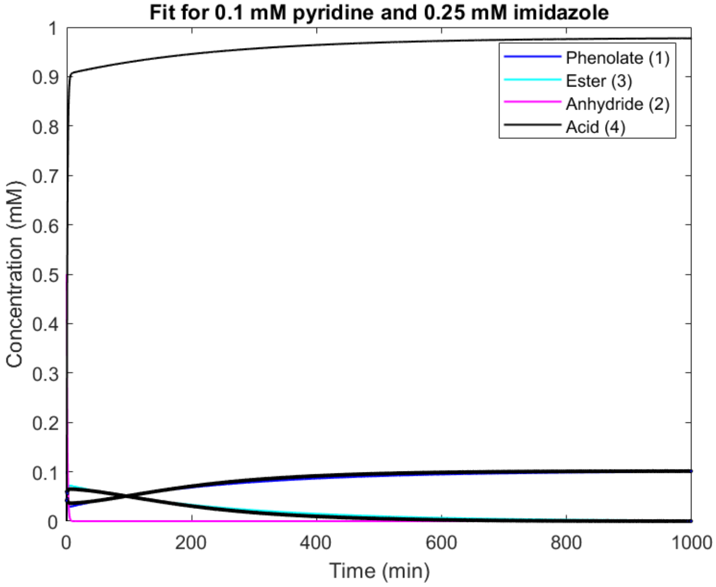

***k*-values**	
k_0	0.0001 min^{-1}
k_1	0.1875 $mM^{-1}min^{-1}$
k_3	0.2762 min^{-1}
k_{cat1}	0.0199 $mM^{-1}min^{-1}$
k_{cat2}	16.52 $mM^{-2}min^{-1}$
k_{cat3}	0.031 $mM^{-2}min^{-1}$
k_{cat4}	0.1321 $mM^{-1}min^{-1}$
k_{cat5}	0.0046 $mM^{-1}min^{-1}$
k_{cat6}	3.0617 $mM^{-1}min^{-1}$

Figure S22: Concentration profiles of different species (phenolate 1, ester 3, acetic anhydride 2 and acetic acid 4) over time for the ester formation with pyridine (0.1 mM) and imidazole (0.25 mM): Experimental data (black dots) and model (lines).

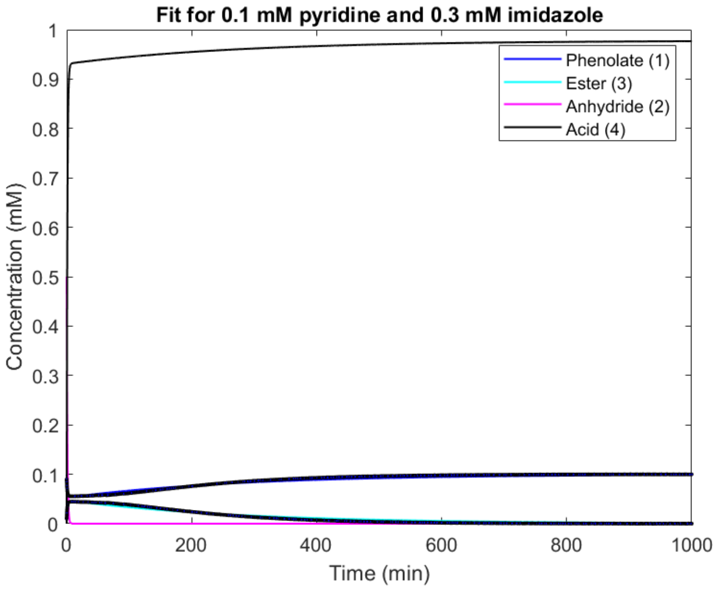

***k*-values**	
k_0	0.0002 min^{-1}
k_1	0.1883 mM^{-1}min^{-1}
k_3	0.302min^{-1}
k_{cat1}	0.0127 mM^{-1}min^{-1}
k_{cat2}	10.1817 mM^{-2}min^{-1}
k_{cat3}	0.0329 mM^{-2}min^{-1}
k_{cat4}	0.1744 mM^{-1}min^{-1}
k_{cat5}	0.0032 mM^{-1}min^{-1}
k_{cat6}	5.2003 mM^{-1}min^{-1}

Figure S23: Concentration profiles of different species (phenolate 1, ester 3, acetic anhydride 2 and acetic acid 4) over time for the ester formation with pyridine (0.1 mM) and imidazole (0.3 mM): Experimental data (black dots) and model (lines).

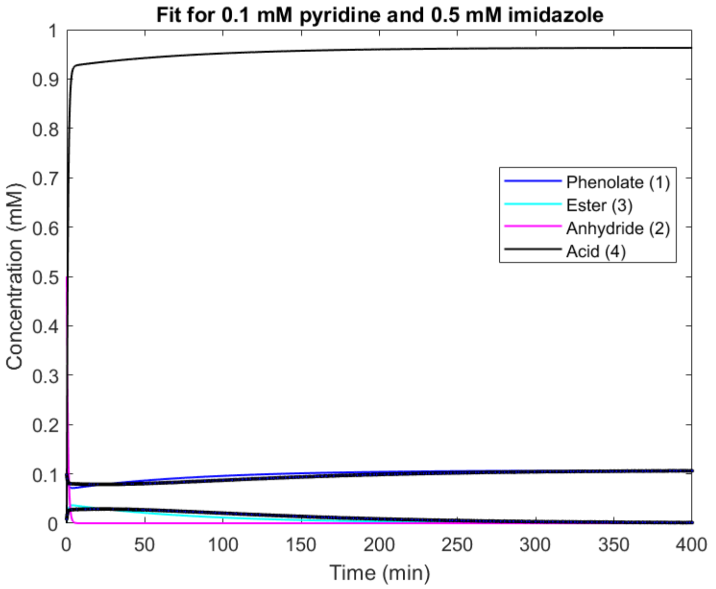

***k*-values**	
k_0	0.0001 min^{-1}
k_1	0.1875 mM^{-1}min^{-1}
k_3	0.4438 min^{-1}
k_{cat1}	0.0295 mM^{-1}min^{-1}
k_{cat2}	8.75 mM^{-2}min^{-1}
k_{cat3}	0.031 mM^{-2}min^{-1}
k_{cat4}	0.2258 mM^{-1}min^{-1}
k_{cat5}	0.0013 mM^{-1}min^{-1}
k_{cat6}	6.1 mM^{-1}min^{-1}

Figure S24: Concentration profiles of different species (phenolate 1, ester 3, acetic anhydride 2 and acetic acid 4) over time for the ester formation with pyridine (0.1 mM) and imidazole (0.5 mM): Experimental data (black dots) and model (lines).

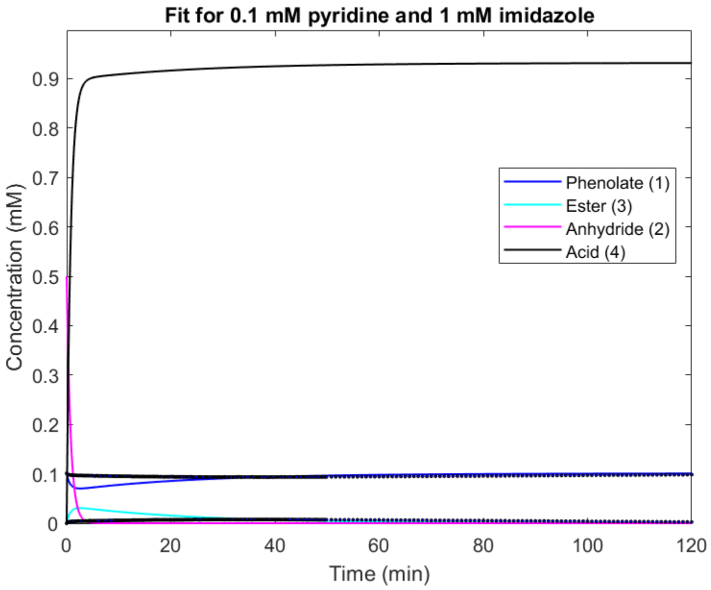

k-values	
k_0	0.0002 min^{-1}
k_1	0.1875 $mM^{-1}min^{-1}$
k_3	0.4438 min^{-1}
k_{cat1}	0.0508 $mM^{-1}min^{-1}$
k_{cat2}	8.75 $mM^{-2}min^{-1}$
k_{cat3}	0.031 $mM^{-2}min^{-1}$
k_{cat4}	0.2258 $mM^{-1}min^{-1}$
k_{cat5}	0.0046 $mM^{-1}min^{-1}$
k_{cat6}	6.1 $mM^{-1}min^{-1}$

Figure S25: Concentration profiles of different species (phenolate 1, ester 3, acetic anhydride 2 and acetic acid 4) over time for the ester formation with pyridine (0.1 mM) and imidazole (1 mM): Experimental data (black dots) and model (lines).

Increasing pyridine concentration (0.1 mM → 0.5 mM→ 1 mM)

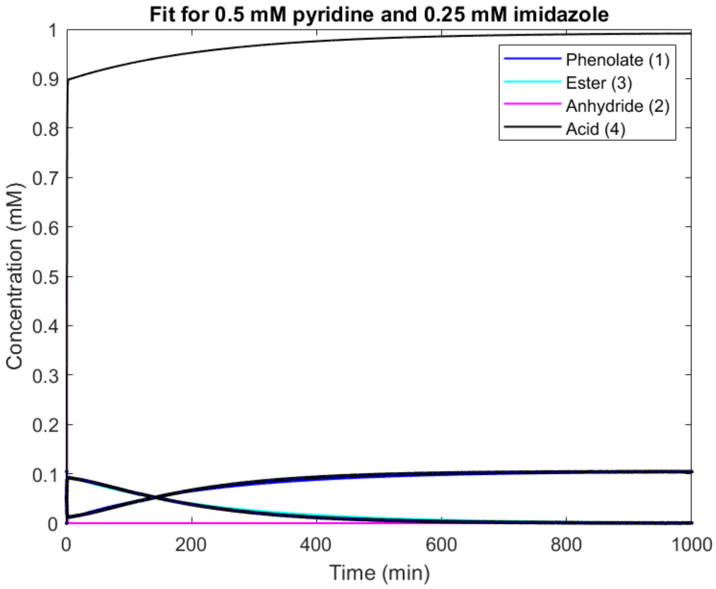

k-values	
k_0	0.0001 min^{-1}
k_1	0.7373 $mM^{-1}min^{-1}$
k_3	0.4341 min^{-1}
k_{cat1}	0.0186 $mM^{-1}min^{-1}$
k_{cat2}	27.8313 $mM^{-2}min^{-1}$
k_{cat3}	0.1221 $mM^{-2}min^{-1}$
k_{cat4}	0.2258 $mM^{-1}min^{-1}$
k_{cat5}	0.0011 $mM^{-1}min^{-1}$
k_{cat6}	3.8786 $mM^{-1}min^{-1}$

Figure S26: Concentration profiles of different species (phenolate 1, ester 3, acetic anhydride 2 and acetic acid 4) over time for the ester formation with pyridine (0.5 mM) and imidazole (0.25 mM): Experimental data (black dots) and model (lines).

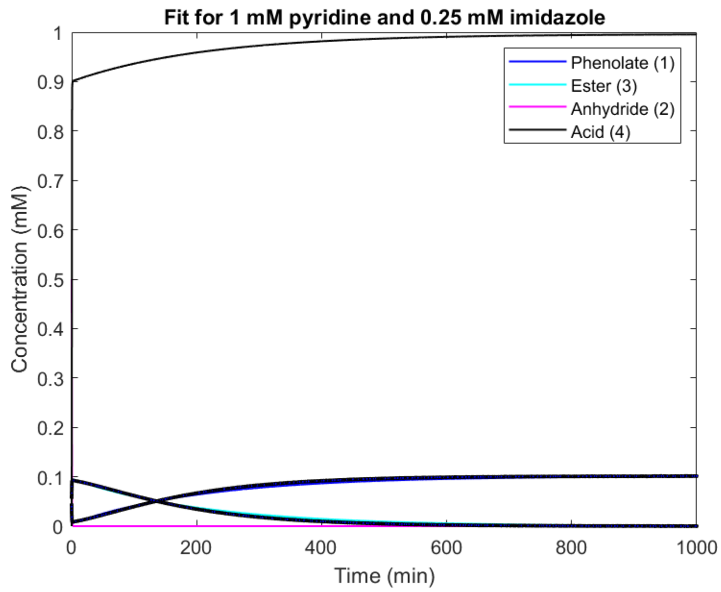

k-values	
k_0	0.0001 min^{-1}
k_1	0.75 $mM^{-1}min^{-1}$
k_3	0.4345 min^{-1}
k_{cat1}	0.0175 $mM^{-1}min^{-1}$
k_{cat2}	35 $mM^{-2}min^{-1}$
k_{cat3}	0.124 $mM^{-2}min^{-1}$
k_{cat4}	0.2258 $mM^{-1}min^{-1}$
k_{cat5}	0.0011 $mM^{-1}min^{-1}$
k_{cat6}	4.5055 $mM^{-1}min^{-1}$

Figure S27: Concentration profiles of different species (phenolate 1, ester 3, acetic anhydride 2 and acetic acid 4) over time for the ester formation with pyridine (1 mM) and imidazole (0.25 mM): Experimental data (black dots) and model (lines).

Decreasing anhydride concentration (0.5 mM → 0.25 mM)

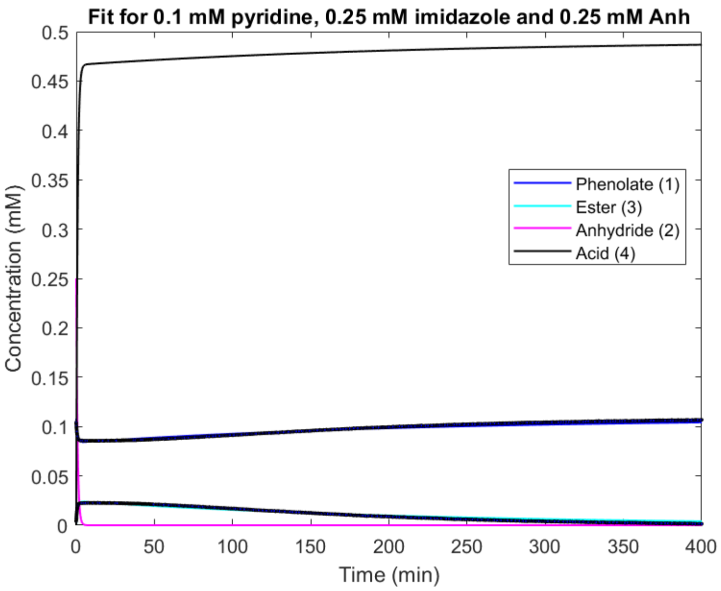

k-values	
k_0	0.0001 min^{-1}
k_1	0.1893 $mM^{-1}min^{-1}$
k_3	0.4438 min^{-1}
k_{cat1}	0.0218 $mM^{-1}min^{-1}$
k_{cat2}	10.4851 $mM^{-2}min^{-1}$
k_{cat3}	0.0314 $mM^{-2}min^{-1}$
k_{cat4}	0.2258 $mM^{-1}min^{-1}$
k_{cat5}	0.0012 $mM^{-1}min^{-1}$
k_{cat6}	6.1 $mM^{-1}min^{-1}$

Figure S28: Concentration profiles of different species (phenolate 1, ester 3, acetic anhydride 2 and acetic acid 4) over time for the ester formation with pyridine (0.1 mM), imidazole (0.25 mM) and acetic anhydride (0.25 mM): Experimental data (black dots) and model (lines).

Increasing anhydride concentration (0.5 mM → 0.75 mM)

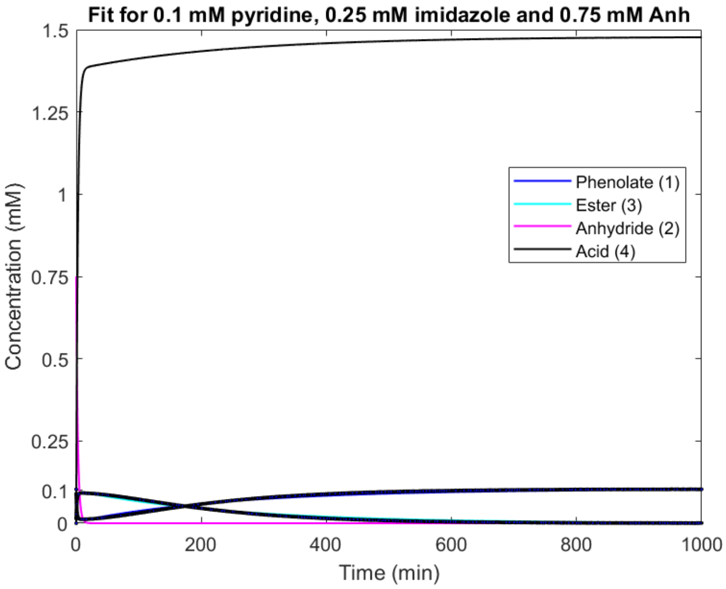

***k*-values**	
k_0	0.0001 min^{-1}
k_1	0.3313 $mM^{-1}min^{-1}$
k_3	0.1116 min^{-1}
k_{cat1}	0.0191 $mM^{-1}min^{-1}$
k_{cat2}	11.2411 $mM^{-2}min^{-1}$
k_{cat3}	0.053 $mM^{-2}min^{-1}$
k_{cat4}	0.0571 $mM^{-1}min^{-1}$
k_{cat5}	0.0012 $mM^{-1}min^{-1}$
k_{cat6}	2.268 $mM^{-1}min^{-1}$

Figure S29: Concentration profiles of different species (phenolate 1, ester 3, acetic anhydride 2 and acetic acid 4) over time for the ester formation with pyridine (0.1 mM), imidazole (0.25 mM) and acetic anhydride (0.75 mM): Experimental data (black dots) and model (lines).

9.4.1 Pyridine variation: experimental data versus model

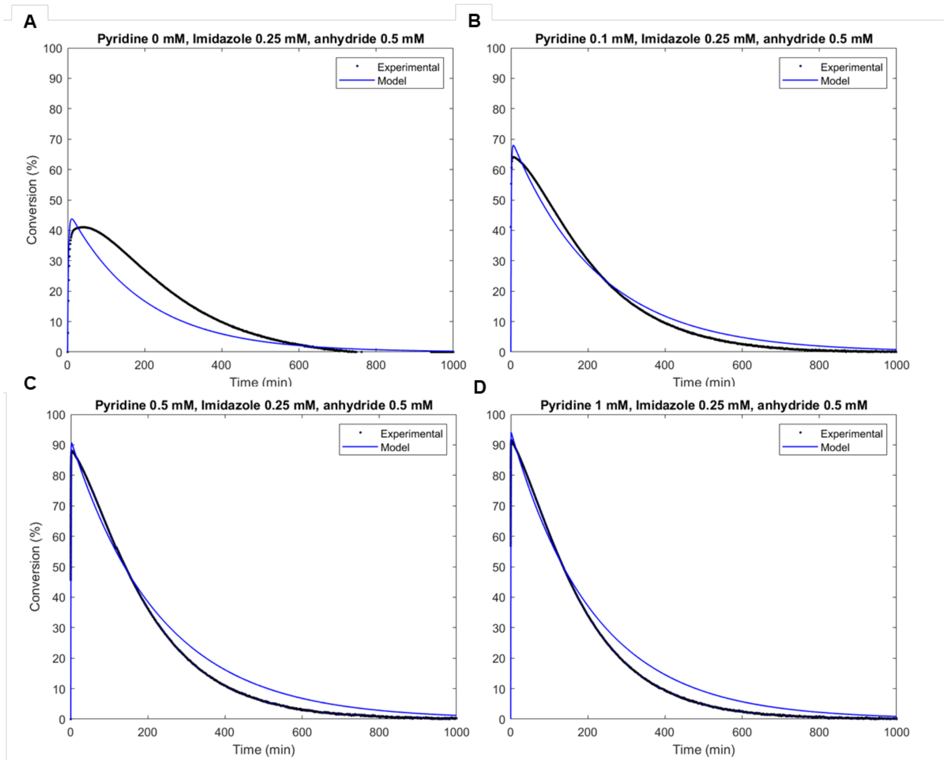

Figure S30: Experimental and model data comparison for varying pyridine 1c concentrations, showing the conversion of *p*-nitrophenol(ate) 1 (start: 0.1 mM) in MOPS buffer (100 mM, pH 7.5) with 5% acetonitrile: (A). 0.5 mM acetic anhydride 2, 0.25 mM imidazole and 0 mM pyridine. (B). 0.5 mM acetic anhydride 2, 0.25 mM imidazole and 0.1 mM pyridine. (C). 0.5 mM acetic anhydride 2, 0.25 mM imidazole and 0.5 mM pyridine. (D). 0.5 mM acetic anhydride 2, 0.25 mM imidazole and 1 mM pyridine. The presented experimental data are equal to Figure 1A.

9.4.2 Imidazole variation: experimental data versus model

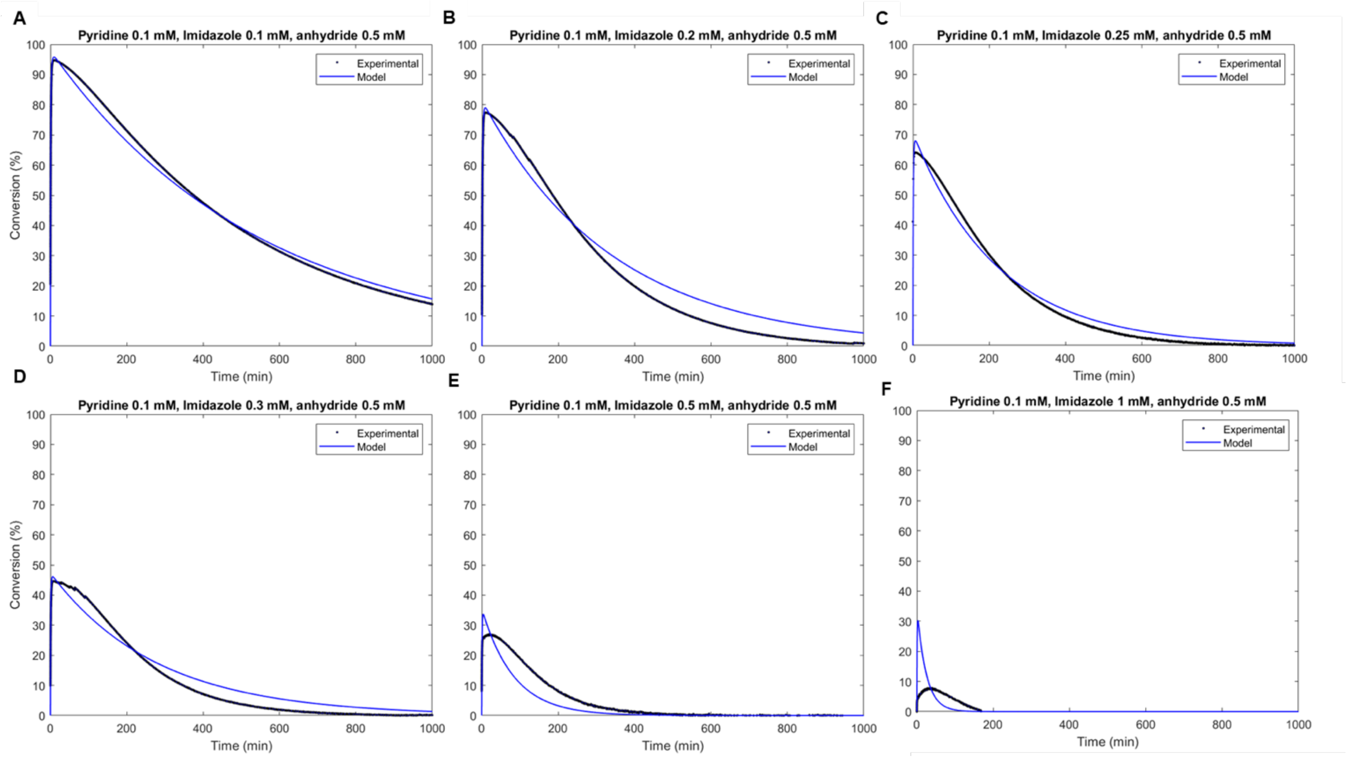

Figure S31: Experimental and model data comparison for varying imidazole 1d concentrations, showing the conversion of *p*-nitrophenol(ate) 1 (start: 0.1 mM) in MOPS buffer (100 mM, pH 7.5) with 5% acetonitrile: (A). 0.5 mM acetic anhydride 2, 0.1 mM imidazole and 0.1 mM pyridine. (B). 0.5 mM acetic anhydride 2, 0.2 mM imidazole and 0.1 mM pyridine. (C). 0.5 mM acetic anhydride 2, 0.25 mM imidazole and 0.1 mM pyridine. (D). 0.5 mM acetic anhydride 2, 0.3 mM imidazole and 0.1 mM pyridine. (E). 0.5 mM acetic anhydride 2, 0.5 mM imidazole and 0.1 mM pyridine. (F). 0.5 mM acetic anhydride 2, 1 mM imidazole and 0.1 mM pyridine. The presented experimental data are equal to Figure 1B.

9.4.3 Acetic anhydride variation: experimental data versus model

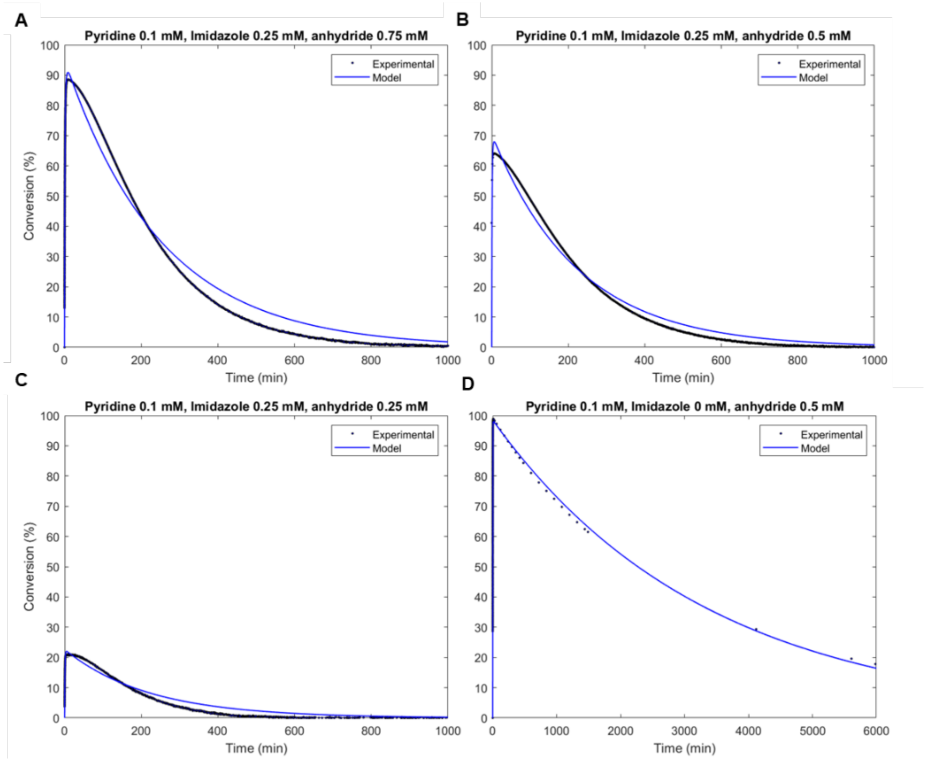

Figure S32: Experimental and model data comparison for varying acetic anhydride 2 concentrations and imidazole, showing the conversion of *p*-nitrophenol(ate) 1 (start: 0.1 mM) in MOPS buffer (100 mM, pH 7.5) with 5% acetonitrile: (A). 0.75 mM acetic anhydride 2, 0.25 mM imidazole and 0.1 mM pyridine. (B). 0.5 mM acetic anhydride 2, 0.25 mM imidazole and 0.1 mM pyridine. (C). 0.25 mM acetic anhydride 2, 0.25 mM imidazole and 0.1 mM pyridine. (D). 0.5 mM acetic anhydride 2, 0 mM imidazole and 0.1 mM pyridine. The presented experimental data are equal to Figure 1C.

9.4.4 Explanation for model optimization and deviations

The k-values were allowed to be optimised by Matlab using the constrained optimization function (fmincon) with a least squared cost function (opposing lower and upper bounds for the k-values: $k_{min}=0.5 \cdot k_0$ and $k_{max}=2 \cdot k_0$). Yet even with the optimization, these k-values show some deviation (Table S3):

Table S3: Final reaction rate constants as determined from experimental data and subsequent Matlab fitting.

k-value	Units	Reaction	Average	St. Dev.	Final
k_0	$(\cdot 10^{-4})$ min^{-1}	Ester hydrolysis uncatalysed	1.23	0.44	1.23 ± 0.44
k_1	$(\cdot 10^{-1})$ $mM^{-1}min^{-1}$	Ester formation uncatalysed	3.57	2.03	3.57 ± 2.03
k_3	$(\cdot 10^{-1})$ min^{-1}	Anhydride hydrolysis uncatalysed	3.00	1.33	3.00 ± 1.33
k_{cat1}	$(\cdot 10^{-2})$ $mM^{-1}min^{-1}$	Ester hydrolysis imidazole catalysed	2.23	1.06	2.23 ± 1.06
k_{cat2}	$(\cdot 10^{1})$ $mM^{-2}min^{-1}$	Ester formation pyridine catalysed	1.88	1.04	1.88 ± 1.04
k_{cat3}	$(\cdot 10^{-2})$ $mM^{-2}min^{-1}$	Ester formation imidazole catalysed	6.41	4.15	6.41 ± 4.15
k_{cat4}	$(\cdot 10^{-1})$ $mM^{-1}min^{-1}$	Anhydride hydrolysis imidazole catalysed	1.66	0.76	1.66 ± 0.76
k_{cat5}	$(\cdot 10^{-3})$ $mM^{-1}min^{-1}$	Ester hydrolysis pyridine catalysed	2.68	1.64	2.68 ± 1.64
k_{cat6}	$mM^{-1}min^{-1}$	Anhydride hydrolysis pyridine catalysed	4.03	1.68	4.03 ± 1.68

The k-values with most variation are related to the anhydride species and a deviation is anticipated, since the acetic anhydride hydrolysis is very rapid [3] and already occurs before the first sample has been measured. Besides, the mechanism for imidazole and pyridine catalysis is in fact more complicated than was assumed in this simplified model and deals with a pre-equilibrium of reactive intermediate formation (acetyl-pyridinium and acetyl-imidazole), acetate ion inhibition and contributions from nucleophilic and general acid/ base catalysis [1b, 6]. Finally, the reactive intermediates for imidazole and pyridine catalysis can also be interconverted, complicating the model.

9.4.5 Ester forward and backward reaction rate comparison

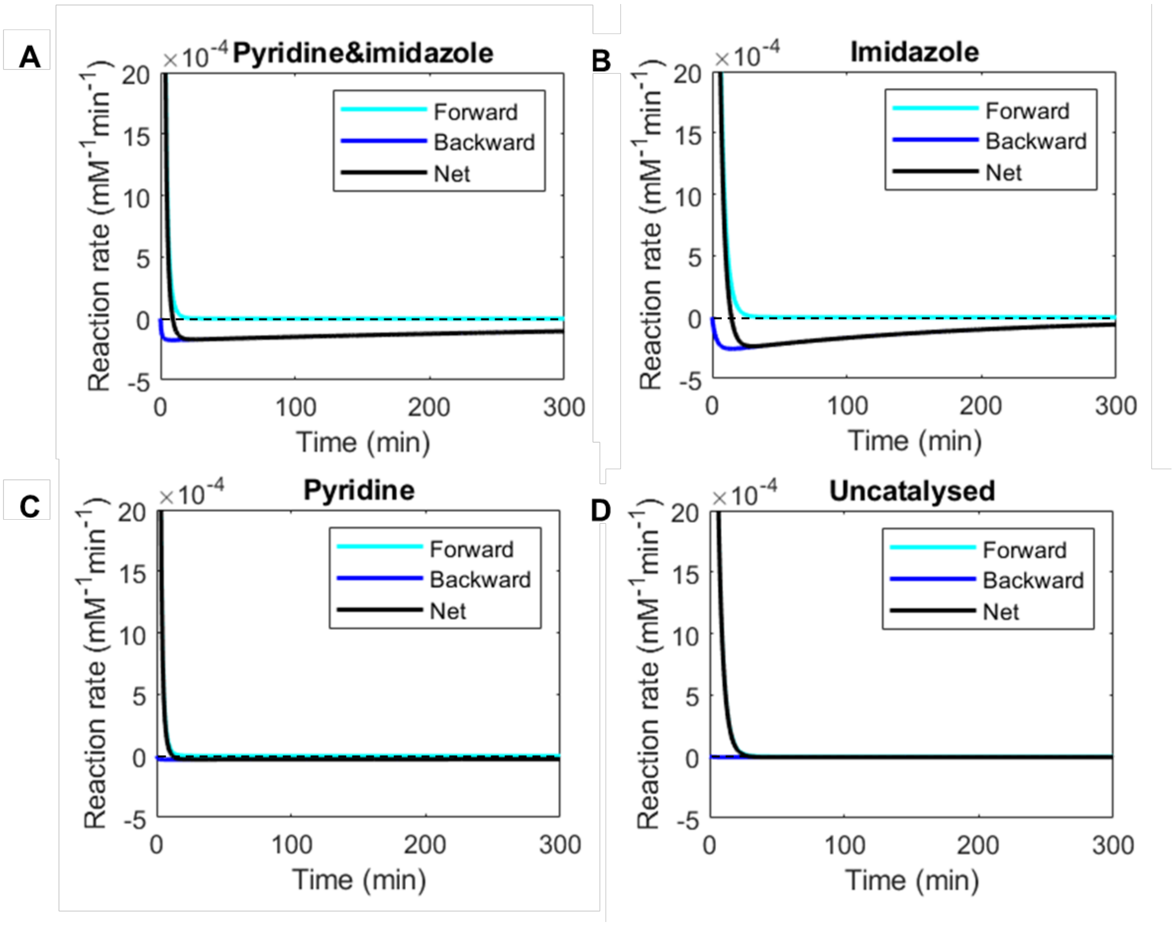

Figure S33: Rate of ester 3 formation (Forward - cyan line), degradation (Backward - blue line) and net formation (Net – black line) based on the rate equations of the kinetic model for various catalytic conditions from 0-300 min: (A). Pyridine and imidazole catalysis: 0.5 mM acetic anhydride 2, 0.1 mM imidazole and 0.1 mM pyridine. (B). Imidazole catalysis: 0.5 mM acetic anhydride 2, 0.25 mM imidazole and no pyridine. (C) Pyridine catalysis: 0.5 mM acetic anhydride 2, 0.1 mM pyridine and no imidazole. (D). Uncatalyzed blank reaction: 0.5 mM acetic anhydride 2. N.B. for panel (D) the forward reaction rate coincides with the net rate and is therefore obscured.

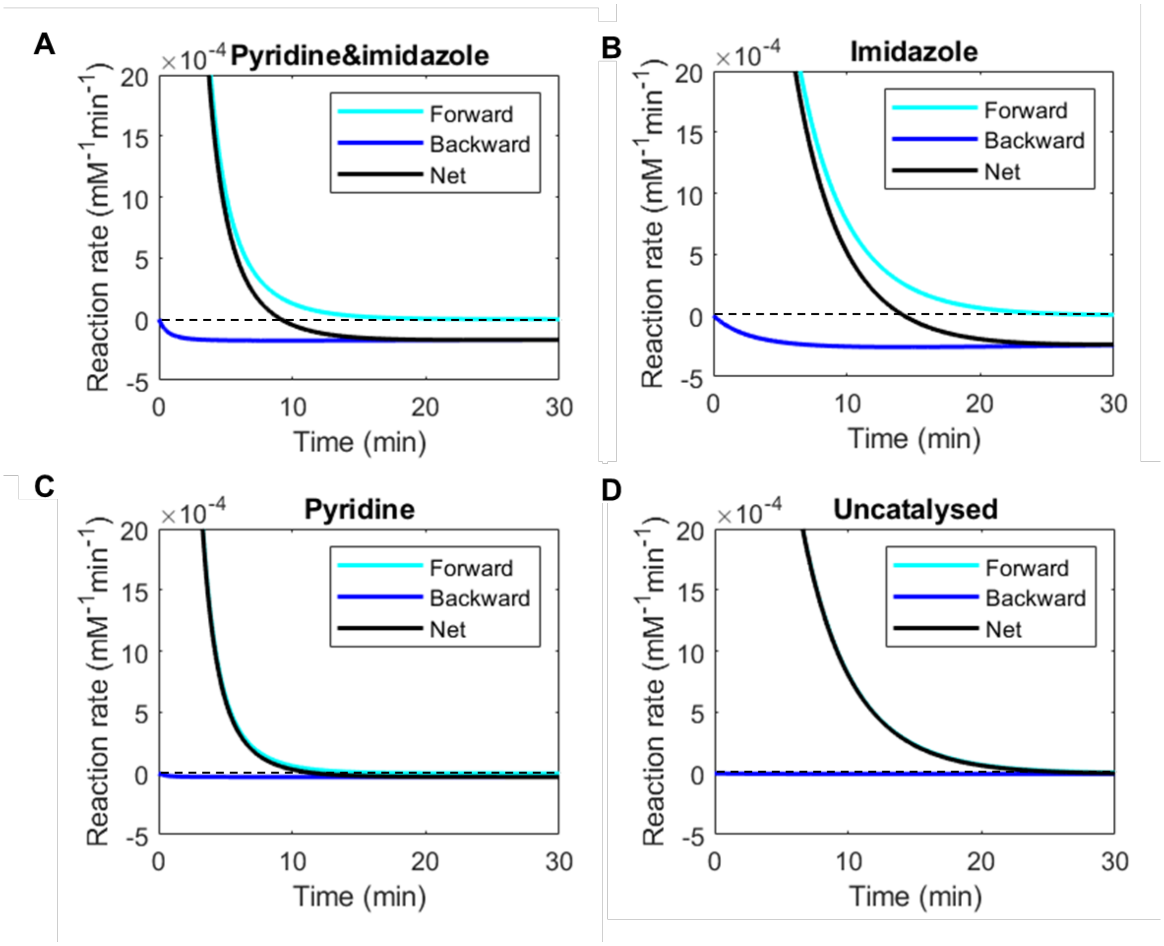

Figure S34: Rate of ester 3 formation (Forward - cyan line), degradation (Backward - blue line) and net formation (Net – black line) based on the rate equations of the kinetic model for various catalytic conditions from 0-30 min: (A). Pyridine and imidazole catalysis: 0.5 mM acetic anhydride 2, 0.1 mM imidazole and 0.1 mM pyridine. (B). Imidazole catalysis: 0.5 mM acetic anhydride 2, 0.25 mM imidazole and no pyridine. (C) Pyridine catalysis: 0.5 mM acetic anhydride 2, 0.1 mM pyridine and no imidazole. (D). Uncatalyzed blank reaction: 0.5 mM acetic anhydride 2. N.B. for panel (D) the forward reaction rate coincides with the net rate and is therefore obscured.

10 FTIR PAANY

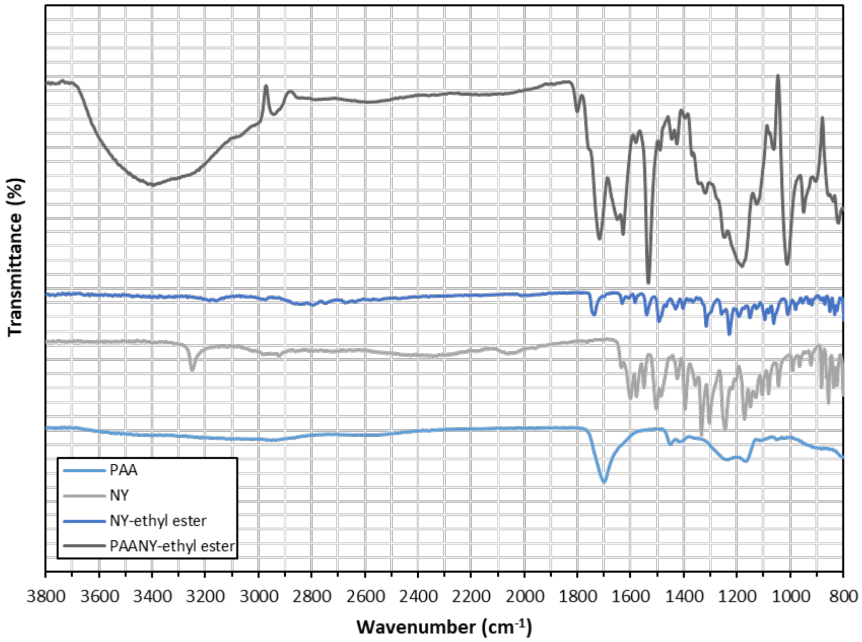

Figure S35: FTIR spectra comparison between PAANY-ethyl ester, PAA, NY-ethyl ester and NY. The following peaks can be identified: C=O stretching mode 1730 cm^{-1} from the ester (in PAANY-ethyl ester and NY-ethyl ester), C=O stretching mode 1700 cm^{-1} from the acid backbone (in PAANY-ethyl ester and PAA), C=O stretching mode 1680 cm^{-1} from the amide bond (in PAANY-ethyl ester, NY-ethyl ester and NY), O-H bending mode 1350 cm^{-1} from the phenol (in PAANY-ethyl ester, NY-ethyl ester and NY) and the C-O-C stretching mode 1050 cm^{-1} from the ester (in PAANY-ethyl ester and NY-ethyl ester).

11 ^{1}H NMR PAANY

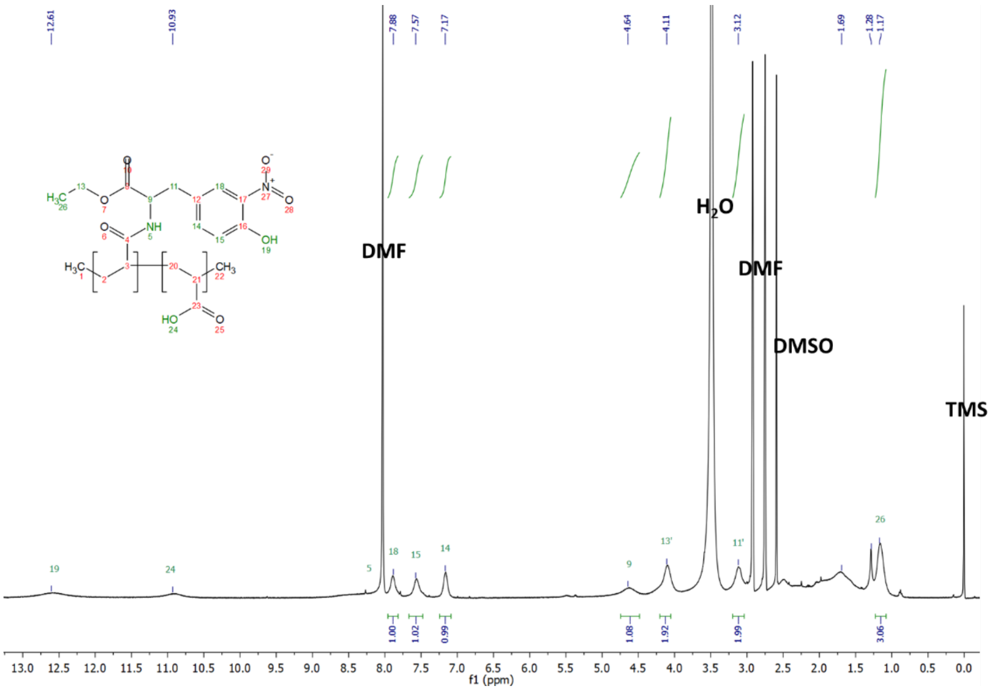

Figure S36: ^{1}H NMR PAANY-ethyl ester in DMF-d7.

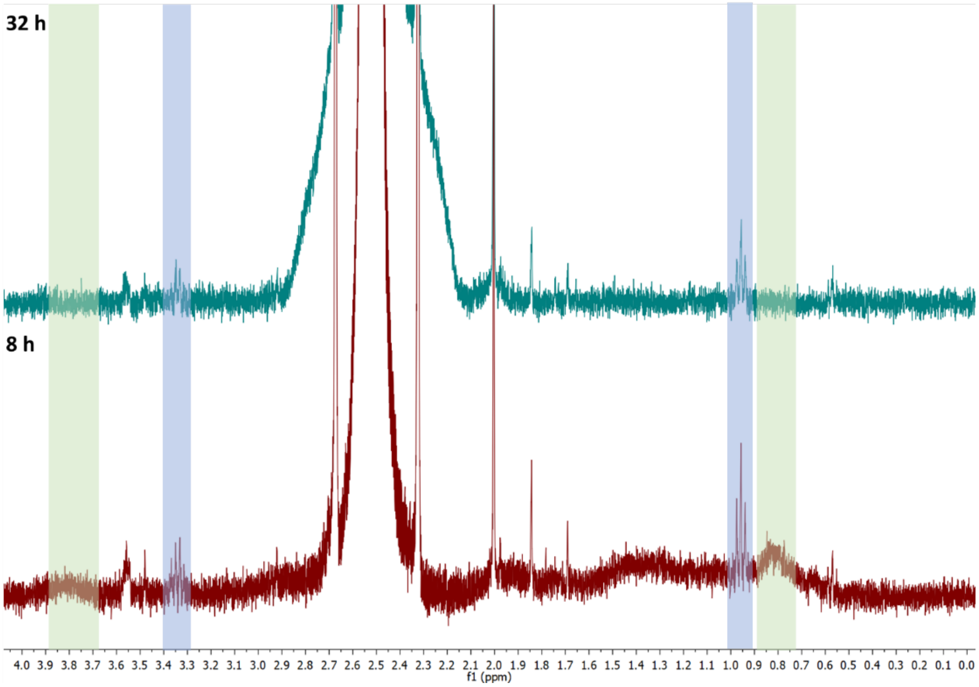

Figure S37: ^{1}H NMR PAANY-ethyl ester hydrolysis in borate buffer (200 mM, pH 8.0), showing the appearance of sharp ethanol peaks around 0.95 ppm (triplet) and 3.35 ppm (quartet) highlighted in the blue frames and the disappearance of the broader polymer ethyl ester peaks (0.8 ppm and 3.8 ppm) highlighted in the green frames.

12 DLS size distributions

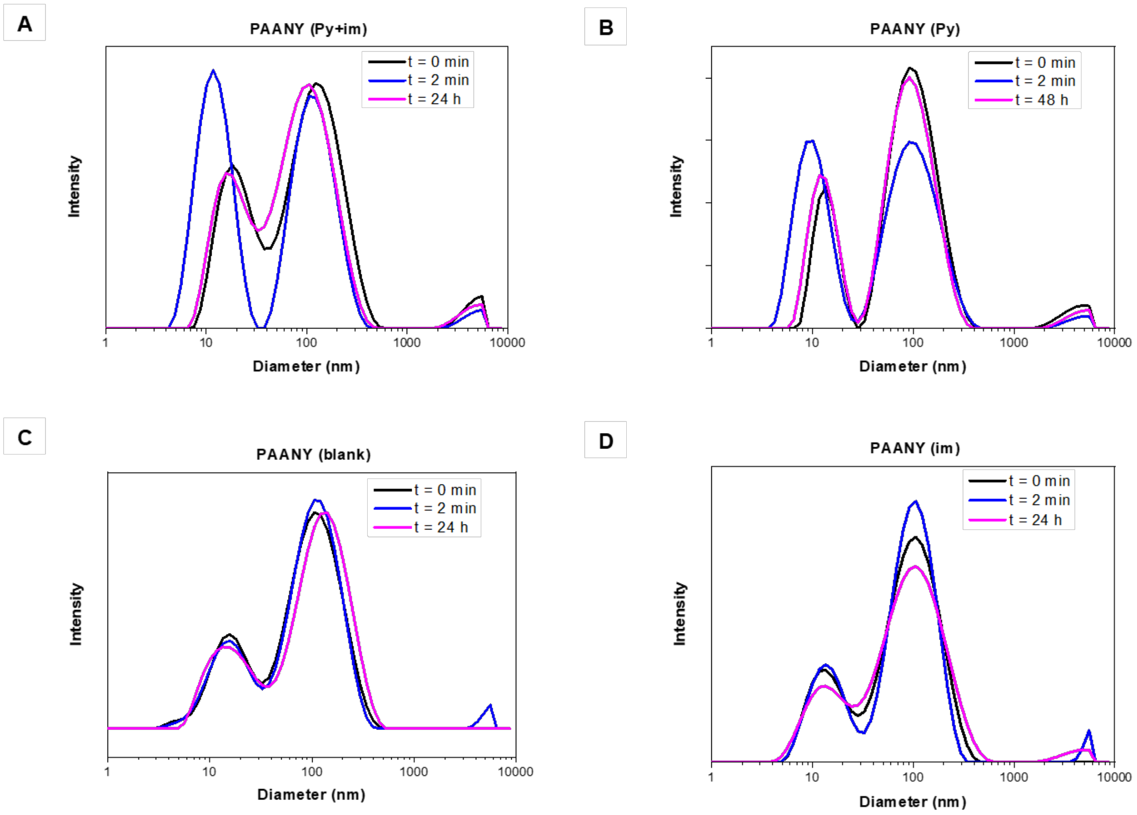

Figure S38: DLS size distributions by intensity, showing the change in size for PAANY in borate buffer (200 mM, pH 8.0). (A). 0.30 mM PAANY (0.24 mg/mL), 6 mM acetic anhydride 2, 0.75 mM imidazole and 0.30 mM pyridine. (B). 0.30 mM PAANY (0.24 mg/mL), 6 mM acetic anhydride 2 and 0.30 mM pyridine. (C). 0.30 mM PAANY (0.24 mg/mL) and 6 mM acetic anhydride 2. (D). 0.30 mM PAANY (0.24 mg/mL), 6 mM acetic anhydride 2 and 0.75 mM imidazole.

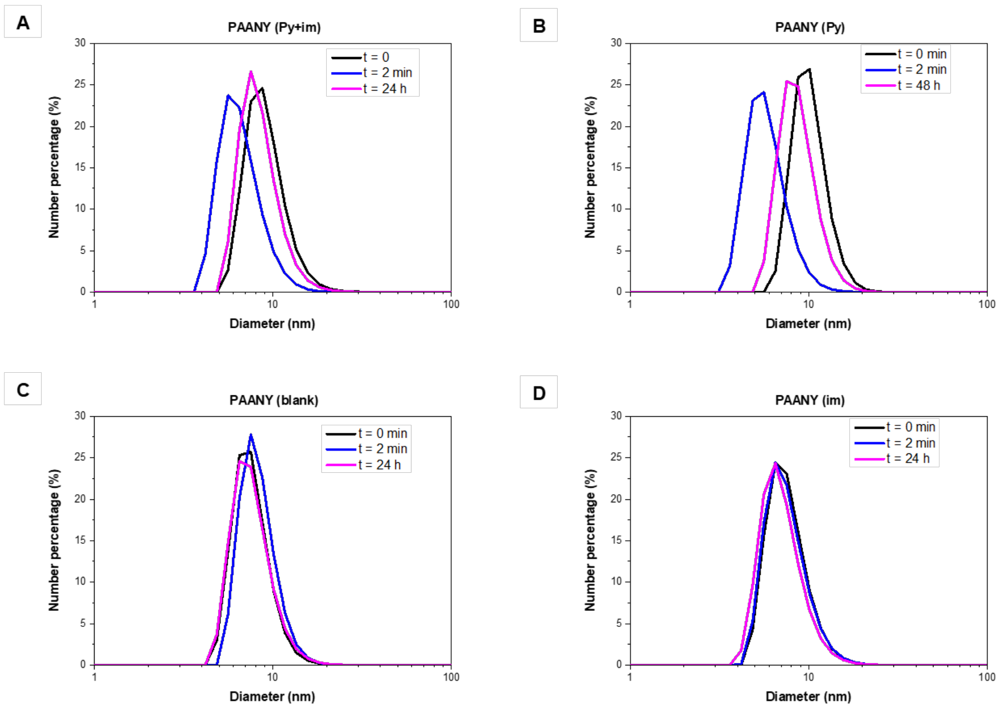

Figure S39: DLS size distributions by number, showing the change in size for PAANY (~10 nm) in borate buffer (200 mM, pH 8.0). (A). 0.30 mM PAANY (0.24 mg/mL), 6 mM acetic anhydride 2, 0.75 mM imidazole and 0.30 mM pyridine. (B). 0.30 mM PAANY (0.24 mg/mL), 6 mM acetic anhydride 2 and 0.30 mM pyridine. (C). 0.30 mM PAANY (0.24 mg/mL) and 6 mM acetic anhydride 2. (D). 0.30 mM PAANY (0.24 mg/mL), 6 mM acetic anhydride 2 and 0.75 mM imidazole.

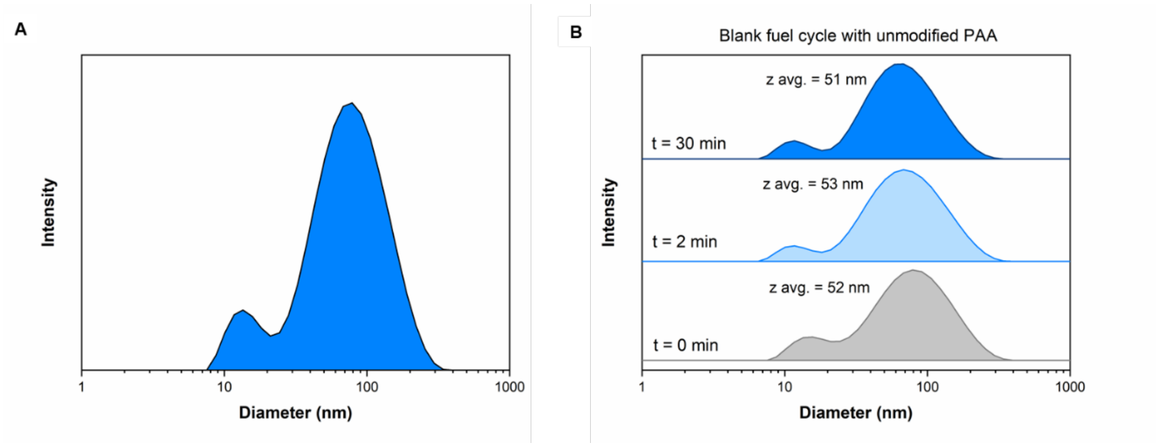

Figure S40: (A) DLS size distribution by intensity of unmodified PAA (0.24 mg/mL) in borate buffer (200 mM, pH 8.0), showing two peaks: a small unimer and larger aggregate peak. (B) DLS size distribution by intensity of blank reaction with unmodified PAA in borate buffer (200 mM, pH 8.0), showing no size change over time. Conditions: PAA (0.24 mg/mL), 6 mM acetic anhydride 2, 0.75 mM imidazole and 0.30 mM pyridine.

13 DLS size calibration

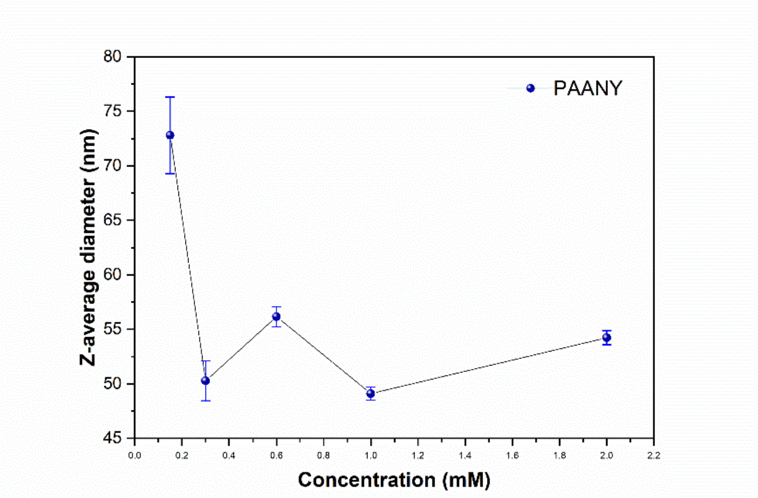

Figure S41: DLS size calibration for various PAANY concentrations in borate buffer (200 mM, pH 8.0). With higher polymer concentrations the size fluctuations become smaller. The reported diameter is the z-average diameter, which takes into account PAANY (~10 nm) and buffer/ polymer aggregates (~100 nm).

14 DOSY diffusion coefficient and size calculation

By measuring the DOSY spectrum of the polymer with NMR and extracting the diffusion coefficient, we can confirm the particle size via the Stokes-Einstein equation:

$$D = \frac{k_B T}{3\pi\mu d}$$

, where *D* is the diffusion coefficient, K_B the Boltzmann constant, *T* the temperature, μ the viscosity of the bulk medium and *d* the solute diameter (polymer).

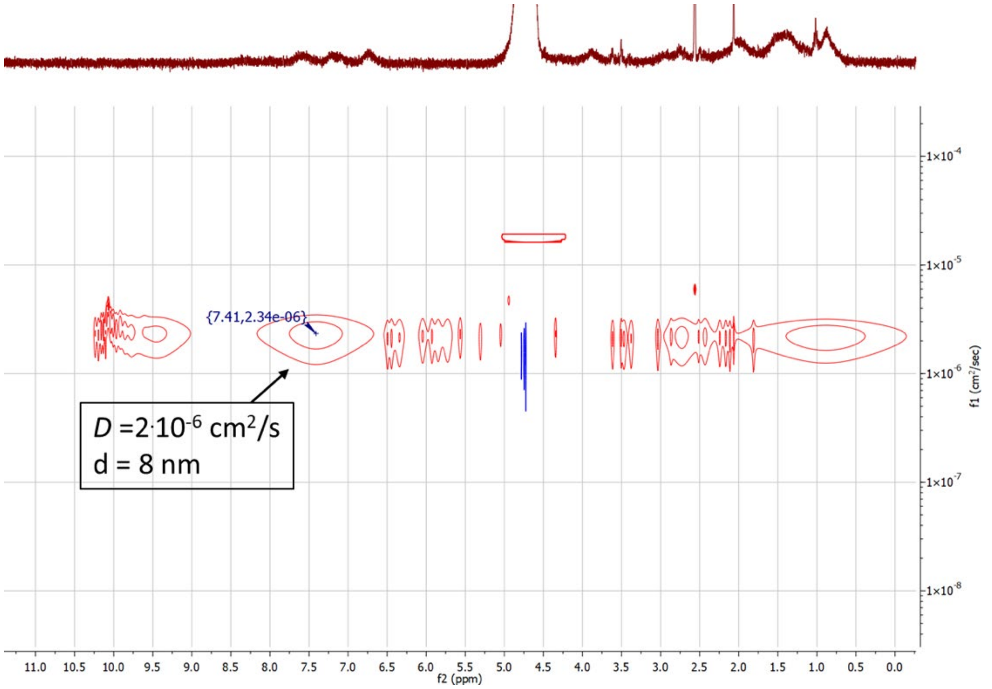

Figure S42: DOSY NMR spectrum of PAANY (0.5 mM – 0.4 mg/mL) in borate buffer (pH 8.0, 200 mM).

15 Cryo-EM imaging of PAANY

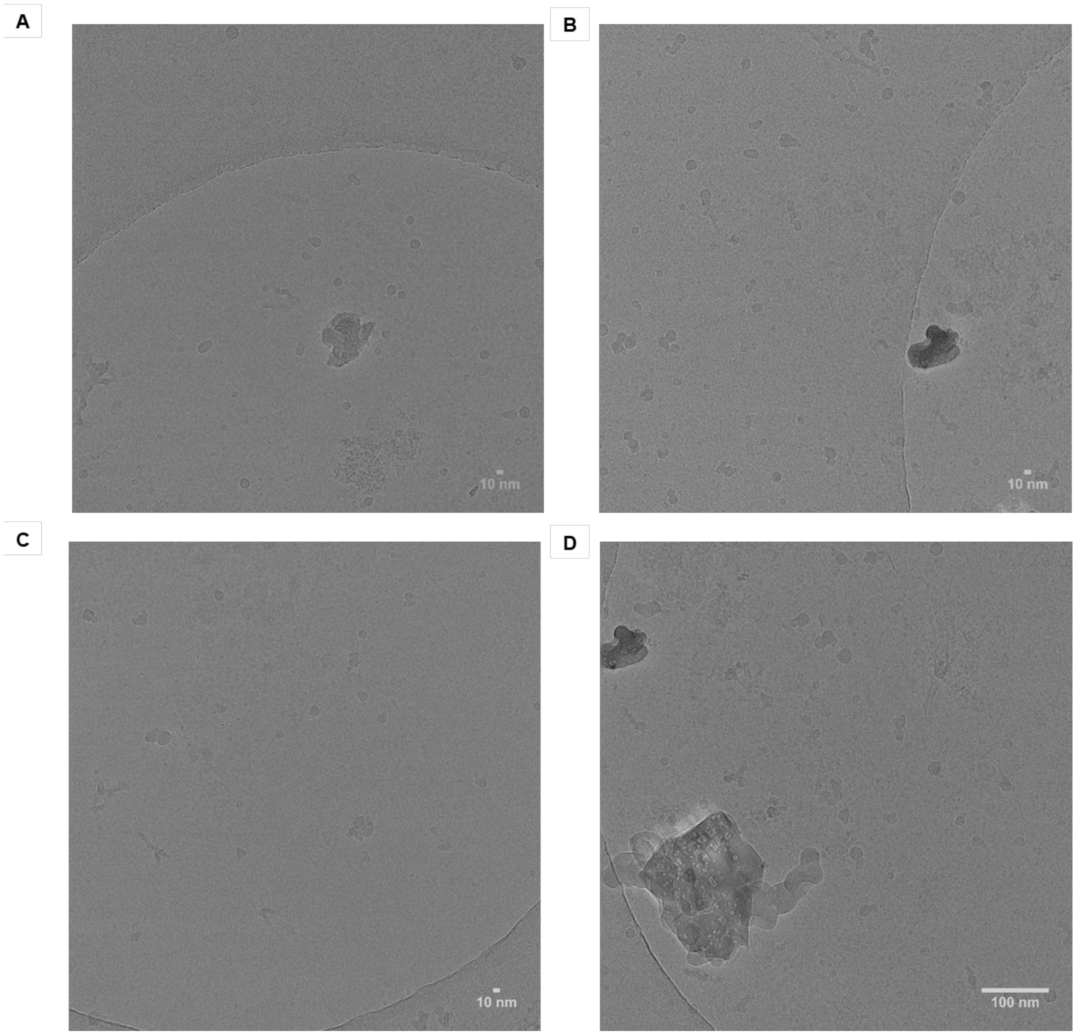

Figure S43: Representative Cryo-EM images of PAANY in borate buffer (200 mM, pH 8.0) at different magnifications. Small spherical structures can be identified with an average diameter of 10 nm along with larger clusters with an average diameter of 24 nm.

16 Viscosity measurements of PAANY

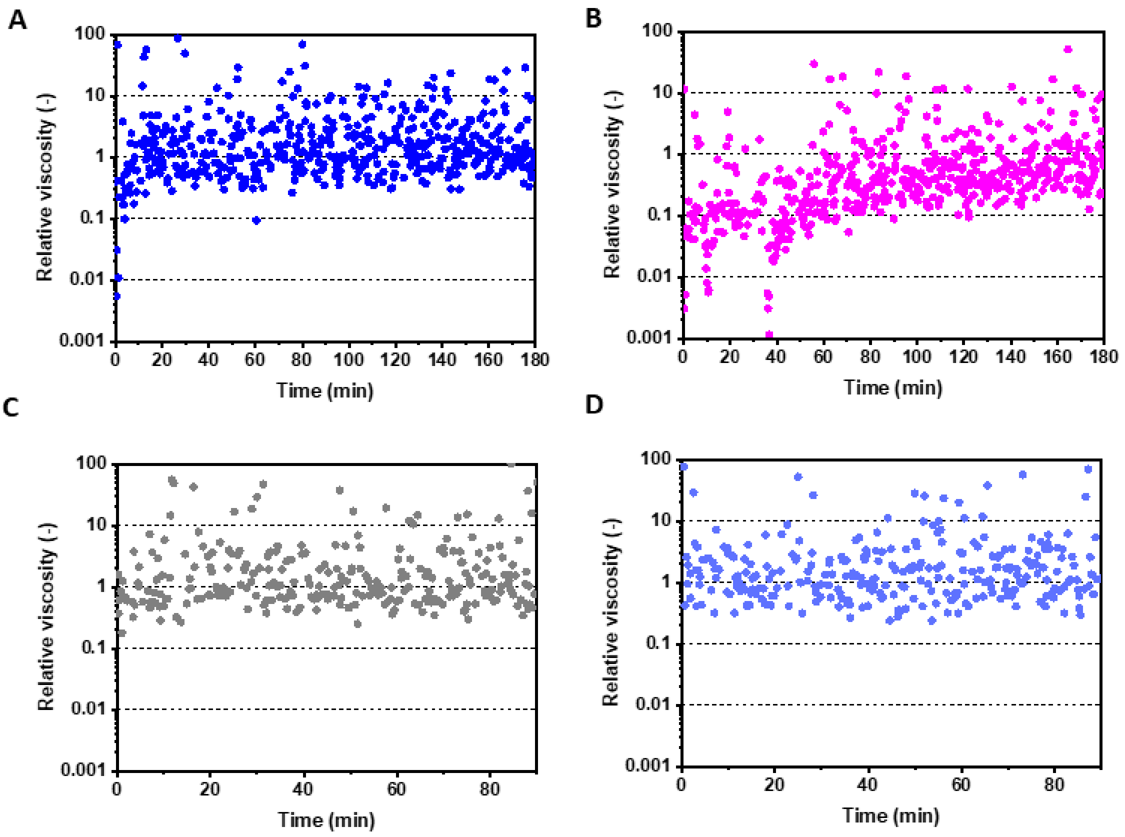

Figure S44: Relative viscosity under time sweep measurements of the following samples: (A). Only PAANY (0.3 mM) in borate buffer 200 mM, pH 8.0. (B). Acetylated PAANY (0.3 mM) with 0.75 mM imidazole, 0.3 mM pyridine, 6 mM acetic anhydride 2 in borate buffer 200 mM, pH 8.0. (C). Only borate buffer 200 mM, pH 8.0. (D). 6 mM acetic anhydride 2 in borate buffer 200 mM. The relative viscosity for each sample was determined by normalisation with the viscosity value at the end of the time sweep measurement. (γ = 0.05%, ω = 1 Hz, 25 °C)

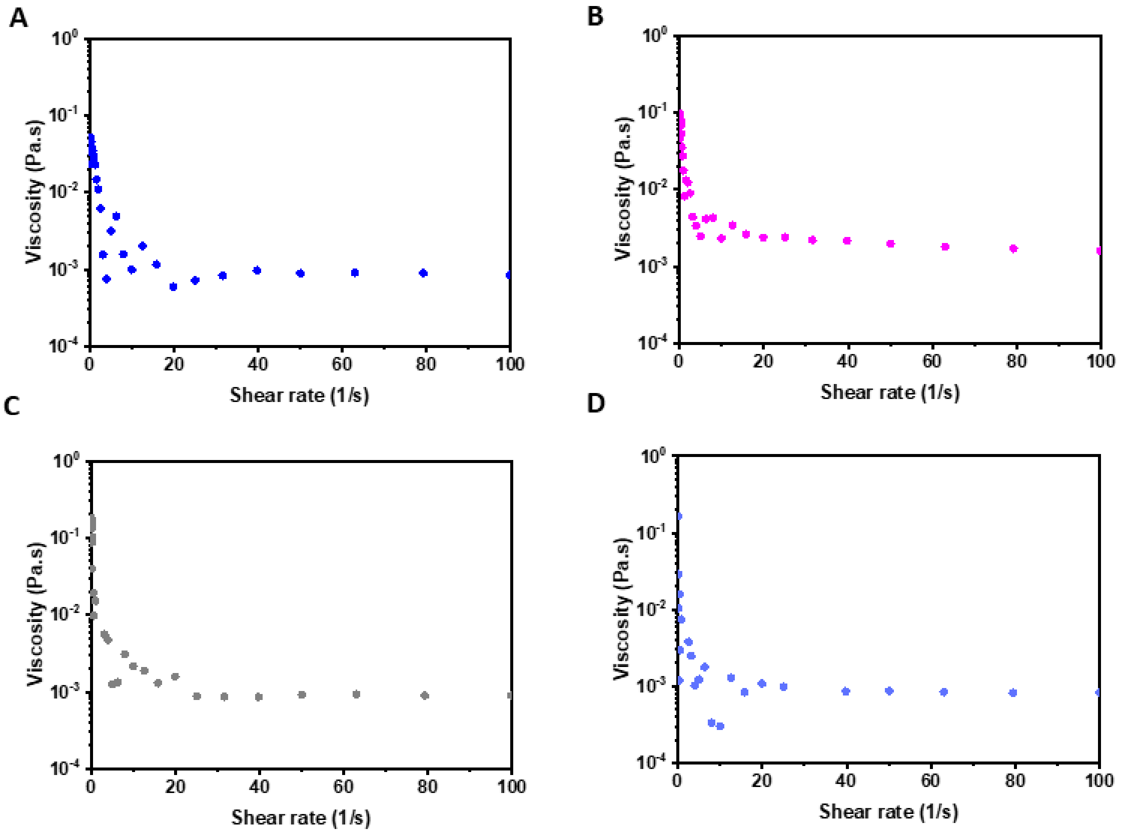

Figure S45: Viscosity under flow step measurements of the following samples: (A). Only PAANY (0.3 mM) in borate buffer 200 mM, pH 8.0. (B). Acetylated PAANY (0.3 mM) with 0.75 mM imidazole, 0.3 mM pyridine, 6 mM acetic anhydride 2 in borate buffer 200 mM, pH 8.0. (C). Only borate buffer 200 mM, pH 8.0. (D). 6 mM acetic anhydride 2 in borate buffer 200 mM.

References

[1] a) J. F. Kirsch, W. P. Jencks, *J. Am. Chem. Soc.* **1964**, *86*, 837-846; b) A. R. Fersht, W. P. Jencks, *J. Am. Chem. Soc.* **1970**, *92*, 5432-5442.

[2] a) J. Bowie, B. Nussey, *J. Mass Spectrom.* **1972**, *6*, 429-442; b) J. H. Bowie, B. J. Stapleton, *Aust. J. Chem.* **1975**, *28*, 1011-1015.

[3] V. Gold, *J. Chem. Soc. Faraday Trans* **1948**, *44*, 506-518.

[4] T. C. Bruice, G. L. Schmir, *J. Am. Chem. Soc.* **1957**, *79*, 1663-1667.

[5] A. Lombardo, *J. Chem. Educ.* **1982**, *59*, 887.

[6] J. F. Kirsch, W. P. Jencks, *J. Am. Chem. Soc.* **1964**, *86*, 833-837.
